# Functional Seasonality of Free-Living and Particle-Associated Prokaryotic Communities in the Coastal Adriatic Sea

**DOI:** 10.3389/fmicb.2020.584222

**Published:** 2020-11-16

**Authors:** Paul A. Steiner, Javier Geijo, Eduard Fadeev, Aleix Obiol, Eva Sintes, Thomas Rattei, Gerhard J. Herndl

**Affiliations:** ^1^Department of Functional and Evolutionary Ecology, University of Vienna, Vienna, Austria; ^2^Department of Microbiology and Ecosystem Science, Division of Computational Systems Biology, University of Vienna, Vienna, Austria; ^3^Institut de Ciències del Mar, Institut de Ci ncies del Mar – Consejo Superior de Investigaciones Cient ficas (ICM-CSIC), Barcelona, Spain; ^4^Instituto Español de Oceanografia, Centre Oceanogràfic de les Balears, Palma, Spain; ^5^Royal Netherlands Institute for Sea Research (NIOZ), Department of Marine Microbiology and Biogeochemistry, Utrecht University, Utrecht, Netherlands

**Keywords:** coastal Mediterranean Sea, seasonal dynamics, eukaryotes, Adriatic Sea, prokaryoplankton, marine snow particles

## Abstract

Marine snow is an important habitat for microbes, characterized by chemical and physical properties contrasting those of the ambient water. The higher nutrient concentrations in marine snow lead to compositional differences between the ambient water and the marine snow-associated prokaryotic community. Whether these compositional differences vary due to seasonal environmental changes, however, remains unclear. Thus, we investigated the seasonal patterns of the free-living and marine snow-associated microbial community composition and their functional potential in the northern Adriatic Sea. Our data revealed seasonal patterns in both, the free-living and marine snow-associated prokaryotes. The two assemblages were more similar to each other in spring and fall than in winter and summer. The taxonomic distinctness resulted in a contrasting functional potential. Motility and adaptations to low temperature in winter and partly anaerobic metabolism in summer characterized the marine snow-associated prokaryotes. Free-living prokaryotes were enriched in genes indicative for functions related to phosphorus limitation in winter and in genes tentatively supplementing heterotrophic growth with proteorhodopsins and CO-oxidation in summer. Taken together, the results suggest a strong influence of environmental parameters on both free-living and marine snow-associated prokaryotic communities in spring and fall leading to higher similarity between the communities, while the marine snow habitat in winter and summer leads to a specific prokaryotic community in marine snow in these two seasons.

## Introduction

Marine snow, described as detrital particles larger than 500 μm, plays an important role in the export of organic carbon to the deep sea and the sequestration via the biological carbon pump (Cho and Azam, 1988; Ducklow et al., 2001; Herndl and Reinthaler, 2013). Chemical characterization, microbial community composition, and activity of marine snow in a large variety of oceanic regions have been extensively studied (Alldredge and Silver, 1988; Simon et al., 2002; Turner, 2002, 2015). However, the majority of studies represent snapshots of the biotic and abiotic characteristics of marine snow captured with sediment traps (De La Rocha and Passow, 2007; Buesseler et al., 2008), marine snow catchers (Riley et al., 2012; van der Jagt et al., 2018), SCUBA divers (Kaltenböck and Herndl, 1992; Müller-Niklas et al., 1994; Rath et al., 1998) or filtration systems (Salazar et al., 2015; Mestre et al., 2017). Only a few studies investigated the dynamics of marine snow and its associated microbial community over a few weeks (Kaltenböck and Herndl, 1992; Müller-Niklas et al., 1994). Recently, the importance of increasing the spatial and temporal resolution of research studies to understand the dynamics in the community composition and functioning of ecosystems has been emphasized (Chow et al., 2013; Fuhrman et al., 2015; Ward et al., 2017). Marine snow-associated microbial communities are considered to be relatively insensitive to changes in environmental parameters of the bulk seawater. With increasing size and volume of the individual marine snow, the physico-chemical conditions determine the composition and activity of the marine snow associated microbiome (Vojvoda et al., 2014; Yung et al., 2016).

Free-living microbial communities are exposed to rapidly changing environmental parameters, resulting in seasonal changes in taxa and closely related ecotypes (Eren et al., 2013; Fuhrman et al., 2015; Yung et al., 2015; Ward et al., 2017; Steiner et al., 2019). Additionally, organic matter leaching from marine snow (Stocker, 2012) links and shapes the free-living community composition (Vojvoda et al., 2014). In contrast, the marine snow-associated microbial community is primarily affected by the origin, composition and developmental stage of the marine snow, and secondarily by the seasonal changes of environmental parameters (Vojvoda et al., 2014; Yung et al., 2016; Duret et al., 2019).

Marine snow represents a specific microhabitat with organic and inorganic nutrient concentrations potentially orders of magnitude higher than in the ambient water (Alldredge and Silver, 1988; Kaltenböck and Herndl, 1992; Müller-Niklas et al., 1994). In addition, it also provides plenty of surfaces for microbial attachment. Therefore, it is not surprising that the prokaryotic community associated with marine snow is distinctly different from that of the ambient water under such conditions (Kaltenböck and Herndl, 1992; Rath et al., 1998; Simon et al., 2002; Simon et al., 2014). However, these reports have been performed during extensive marine snow formation mainly in the summer months of temperate seas. Whether these differences between the free-living and marine snow-associated prokaryotic community composition are maintained throughout the seasonal cycle in temperate seas, however, remains unknown.

The northern Adriatic Sea is a temperate, coastal system with a high abundance of marine snow receiving considerable attention since the 1980’s (Herndl and Peduzzi, 1988; Herndl, 1992; Kaltenböck and Herndl, 1992; Rath et al., 1998). The extent of marine snow formation has been suggested to be due to changes in temperature and nutrient concentrations as well as to the specific water mass circulation patterns in the northern Adriatic Sea (Cozzi et al., 2004). We hypothesized that marine snow offers more stable environmental conditions resulting in a more stable community over the two seasons than the ambient water for the free-living microbial community. Thus, we analyzed the dynamics of the composition of the free-living and particle-associated microbial community based on 16S rRNA gene amplicon sequences over a seasonal cycle in the northern Adriatic Sea. Furthermore, the functional potential of marine snow-associated and free-living prokaryotic community at two contrasting seasons (summer 2015 and winter 2016) was assessed using metagenomics analysis. Finally, the eukaryotic and prokaryotic community composition was related to the environmental parameters and the implications of the prokaryotic metabolic potential for biogeochemical cycles are discussed.

## Materials and Methods

### Sampling

Samples were collected at twelve days covering a 1.5-year period from April 2015 to July 2016 in the coastal northern Adriatic Sea off Rovinj, Croatia (45.08347°N, 13.60518°E). Marine snow was collected with 100 mL sterile syringes at 15 ± 2 m depth by SCUBA diving and processed as described elsewhere (Steiner et al., 2019). In brief, the marine snow collected in several syringes was pooled into a 0.1 M HCl-rinsed glass bottle on deck and stored at *in situ* temperature in the dark. The marine snow included the pore water and a small amount of the water surrounding marine snow. The ambient water was collected at 15 m depth with 5 L Niskin bottles. The samples were transferred to the Center for Marine Research of the Ruder Bošković Institute at Rovinj within 30 min after sample collection. Temperature, salinity and *in vivo* fluorescence representing chlorophyll *a* concentrations were determined monthly with a SBE25 conductivity-temperature-depth probe (Sea-Bird Electronics, WA, United States) at the long-term monitoring station RV001 as described in Ivancic et al. (2018).

### DNA Extraction

Marine snow (150 mL to 500 mL) and 1 L of ambient water were filtered onto 0.2 μm polyethersulfone filters (47 mm diameter, Supor, PALL Gelman) using an aspirator pump (Cole-Parmer). Filters were placed in cryovials (Biozym), flash-frozen in liquid nitrogen and stored at −80°C. DNA was extracted using a standard phenol-chloroform method as described in Steiner et al. (2019). Hereinafter, ambient water and marine snow are abbreviated as ambient water (AW) and marine snow (MS), respectively. Metagenome samples are labeled indicating the season, winter (W) or summer (S), followed by AW or MS, and the duplicate sample number 1 or 2. Duplicates collected in winter (W) correspond to samples collected on 1 and 2 February 2016 and summer (S) duplicates correspond to samples collected on 24 and 25 June 2015. All sequence data are publicly available at the European Nucleotide Archive (ENA) at EMBL-EBI under accession number PRJEB38662^1^. Data was deposited using brokerage service of the German Federation for Biological Data (GFBio; Diepenbroek et al., 2014) in compliance with the Minimal Information about any (x) Sequence (MIxS) standard (Yilmaz et al., 2011).

### Prokaryotic Community Composition Using Amplicon Sequencing

Twelve MS and 11 AW samples were collected for amplicon sequencing of the 16S rRNA gene at the same location as the metagenomics samples. Water samples were collected seasonally over 1.5 years and processed as described elsewhere (Steiner et al., 2019). The primers 341_ill forward (TCGTCGGCAGATGTGTATAAGAGACAGCCTACGGGNGG CWGCAG) and 802_ill reverse (GTCTCGTGGGCTCGGAGA TGTGTATAAGAGACAGGACTACHVGGGTATCTAATCC) targeted the V3–V4 hypervariable region (∼460 bp) of the 16S rRNA of bacteria and archaea. In brief, PCR amplification was performed with the primers 341_ill forward and 802_ill reverse containing adaptors and a KAPAHiFi Mastermix (Peqlab) using the following program: initial denaturation at 94°C for 3 min, followed by 20 cycles of 94°C for 30 s, annealing at 56°C for 30 s and extension at 72°C for 90 s. A final extension step was carried out at 72°C for 7 min, followed by cooling at 4°C. PCR products were purified using Agencourt AMPure XP magnetic particles (Beckman Coulter) and quantified with a Quant-IT PicoGreen^®^ Assay (Invitrogen). Subsequently, Nextera PCR was performed with the same thermocycling conditions as described above for 10 additional cycles, followed by pooling and sequencing on an Illumina MiSeq system (Microsynth AG, Balgach, Switzerland) using v2 chemistry. Amplicon sequences were demultiplexed and trimmed by Microsynth AG (Balgach, Switzerland). All samples were sequenced with a coverage of 1.0 and the number of reads ranged from 7,256 to 6,4801 per sample.

Bioinformatics analysis followed the ‘DADA2 1.12’ (Callahan et al., 2016) pipeline tutorial with specific adaptations when filtering and trimming: truncLen = *c*(225, 210), maxN = 0, minQ = 2, maxEE = *c*(3, 3), truncQ = 0. Singletons, mitochondria and chloroplast sequences were removed. Statistical analysis of the amplicon sequences was conducted using R^2^ in RStudio v.1.2.5033 (RStudio-Team,, 2015) with the package phyloseq v.1.30.0 (McMurdie and Holmes, 2013). The obtained amplicon sequence variants (ASVs) were variance stabilized (McMurdie and Holmes, 2014) and a differential gene expression analysis was conducted using the R package DESeq2 v.1.26.0 (Love et al., 2014). Differences in variance stabilized abundance of ASVs were considered significant if *p* < 0.05. Only ASVs with a log_2_ fold change higher than 1 in sequence abundance were further analyzed.

### Metagenomics Analysis of Prokaryotes

Metagenomic DNA was quantified fluorometrically using the Quant-iT^TM^ PicoGreen^®^ Assay (Invitrogen) on a plate reader (Infinite 200). The library preparation of metagenomic DNA was done with the Westburg kit and sequencing was performed on an Illumina HiSeq 2,500 (2 × 150 paired-end platform) at the Vienna BioCenter Core Facilities GmbH (VBCF). Metagenome sequences were quality checked using fastQC v.0.11.8. Sequences were trimmed with trimmomatic v.0.39 (Bolger et al., 2014) and a sliding window size of 3 bases and a quality of 30. Assembly was done with megahit v.1.1.2 (Li et al., 2015).

The program phyloFlash v.3.3 (Gruber-Vodicka et al., 2019) was used to analyze the prokaryotic community composition based on 16S rRNA obtained from metagenomes with the following settings: -lib LIB -emirge -poscov -treemap. The correlation between the 16S rRNA gene amplicon data matrix and the extracted 16S rRNA genes from the metagenome was tested using Mantel test implemented in the R package vegan v.2.5.6. Prokaryotic OTUs of eight metagenomes were correlated to metataxonomic ASVs determined in the corresponding MS and AW samples collected on 24 and 25 June 2015 and 1 and 3 February 2016.

Further taxonomic and functional analyses were performed with MEGAN Community Edition v.6.17.0 (Huson et al., 2007) with default settings (min score: 50, max expected: 0.01, min percent identity: 0, top percent: 10, min support percent: 0.05, min support: 0, LCA algorithm: naive, percent cover: 100, read assignment mode: readCount). Reads were blasted against the NCBI nr database using DIAMOND with short reads settings: –b 12.0 –k 1 –f 100 –e 0.00001 –p 20. The taxonomic affiliation was determined by aligning the reads to the database prot_acc2tax-Jul2019 × 1.abin. The database acc2eggnog-Oct2016X.abin was used for functional assignment in MEGAN6. Reads putatively assigned to prokaryotes were extracted from the MEGAN6 file using the ‘extract to new document’ function. The prokaryotic reads were visualized using taxonomy and EGGNOG viewer (Powell et al., 2014) of MEGAN6 in absolute comparison mode. Taxonomic and gene assignments to clusters of orthologous groups (COGs) were extracted using the ‘extract to new document’ function to determine the taxonomic affiliation of the COGs. Subsequently, prokaryotes were sorted out from eukaryotic and other reads. The ‘export’ function was used to extract absolute numbers of reads assigned to COGs. Absolute read counts were variance stabilized (McMurdie and Holmes, 2014) and differential gene expression analysis was conducted using the R package DESeq2 v.1.26.0 (Love et al., 2014). COGs were selected for further analysis if differences in their variance-stabilized abundance were significant (*p* < 0.05) and the log_2_ fold change of sequence abundance was > 1. COGs with assignment to transport function were extracted from the variance-stabilized data set and used for further analyses.

Marker gene analysis was performed based on the Kyoto Encyclopedia of Genes and Genomes Orthology (KOs) functional classification of metabolisms (Supplementary Table S1). The presence of 69 selected key marker genes (Acinas et al., 2019) was checked against the aligned metagenomic reads. Predicted genes of aligned reads were determined with MetaProdigal v.2.6.3 (Hyatt et al., 2012) and quantified using FPKM (Fragments Per Kilobase Million) normalization.

### Metagenomics Analysis of Eukaryotes

The eukaryotic community composition was analyzed as described elsewhere Obiol et al. (2020). Briefly, a BLAST search against a subset of the *eukaryotesV4* database with > 90% similarity and > 70% alignment coverage was performed to retrieve fragments of the 18S rRNA V4 region from the metagenomes. The extracted fragments were mapped to the full *eukaryotesV4* database using USEARCH v.11 local alignment at 97% identity. All hits with maximal score for every fragment were selected. All top hits of each read were merged into one, keeping the minimum consensus taxonomic level for taxonomic classification. Reads which resulted in no consensus taxonomic level remained unclassified.

### Analyses of the Structure of the Microbial Community

Diversity indices were calculated using the package vegan v.2.5.6 in R on rarefied data to the lowest number of ASVs. Diversity indices, one-way ANOSIM, PERMANOVA, SIMPER and principal component analysis (PCA) were calculated in PAST3 (Hammer et al., 2001) using Euclidean similarity index. Graphs were created with the program SigmaPlot 14.0 and Venny 2.1 (Oliveros, 2015).

## Results and Discussion

### 16S rRNA Amplicon Sequence Analysis

#### Taxonomic Analysis

On average 891 ± 232 ASVs were hit by 46797 ± 13521 sequences in the AW and 437 ± 219 ASVs were hit by 31548 ± 17230 sequences in MS (Supplementary Figure S1). In total 1711 ASVs had a prevalence of more than 10%, implying the presence of these ASVs in at least two samples.

#### Diversity Indices

Species richness was on average 1.5 ± 0.6 times higher in the AW than MS in spring, summer and fall (Supplementary Table S2 and Supplementary Figure S2). In the winter, however, species richness in the AW was 5.4 ± 6.9 times higher than in MS (Supplementary Table S2 and Supplementary Figure S2). The observed low species richness of MS-associated communities as compared to AW is in agreement with previous findings and was linked to the high nutrient concentrations these microhabitats offer (DeLong et al., 1993; Acinas et al., 1999). The noticeably lower species richness in MS in winter compared to the other seasons and to AW concurred with lower Shannon diversity (3.3 ± 0.2) as compared to the average diversity in MS (4.9 ± 0.4; Supplementary Figure S2). This is likely due to the dominance of *Synechococcus* ASVs in the winter MS communities (Supplementary Figure S3).

#### Prokaryotic Community Analysis

The core community of each month, resembled by the number of prokaryotic classes found in both, the AW and MS, ranged between 20 and 23 in spring, summer and fall. In winter, the core community was made up by a lower number of classes (16 classes) compared to the other seasons (Supplementary Figure S4). However, the number of bacterial classes present exclusively in the AW was relatively high in winter (14 classes as compared to, on average, 4.7 ± 3.4 classes in spring, summer, and fall; Supplementary Figure S4) and comprised 1.4% of all sequences. The largest part thereof was made up by Euryarchaeota, Dadabacteria, and Patescibacteria. These classes contain ecotypes with seasonal patterns, surface water and terrestrial or groundwater clades (Hugoni et al., 2013; Herrmann et al., 2019; Graham and Tully, 2020). The fraction of bacterial classes occurring exclusively in either the AW or MS was on average 0.05 ± 0.04% in spring, summer, fall and in MS in winter, indicating a minor role of habitat specialists in terms of sequence proportions.

**FIGURE 1:**
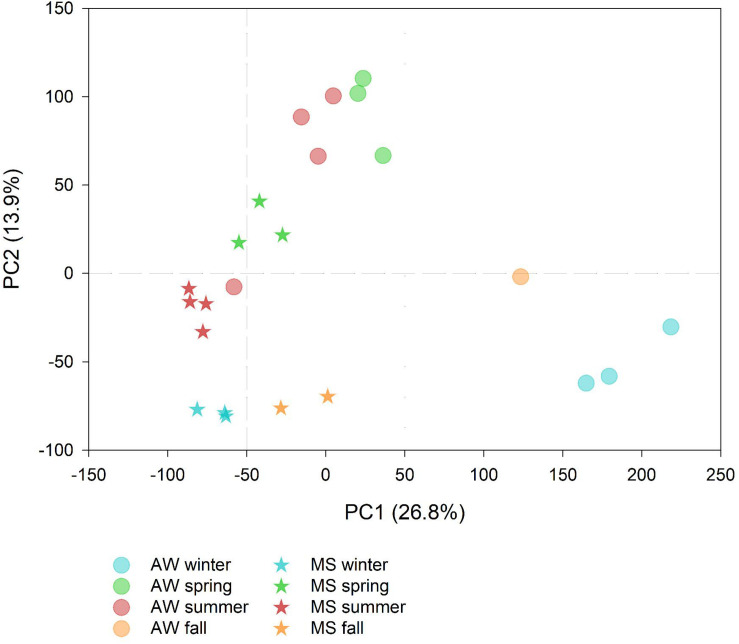
Principal component analysis showing the seasonal differences in the marine snow (MS)-associated and ambient water (AW) prokaryotic community composition based on amplicon sequencing (symbols represent data collected on 12 and 11 days, respectively) labeled according to the season. The percentage of variation explained by each axis is indicated in parenthesis.

Summer-spring communities were distinct from winter-fall prokaryotic communities, with the free-living communities showing larger seasonal dissimilarities than the MS-associated prokaryotic communities (Figure 1). This suggests a more pronounced impact of seasonality on the free-living than on MS-associated prokaryotes possibly due to the more stable microenvironmental conditions in MS than in the AW (Vojvoda et al., 2014; Yung et al., 2016).

#### Prokaryotic Taxa Enriched in Marine Snow and Ambient Water

Overall, the free-living and MS-associated communities differed significantly (one-way ANOSIM *p* < 0.05; Figure 1). Yet, the low number of samples precluded to statistically test whether the free-living and MS-associated communities differed in particular seasons. However, ASVs with significantly different sequence abundances between seasons were obtained. The AW sample collected at 30 July 2016 was identified as an outlier and excluded from the differential sequence abundance analysis (DESeq2) due to high compositional similarity to the summer MS community (Figure 1).

**FIGURE 2:**
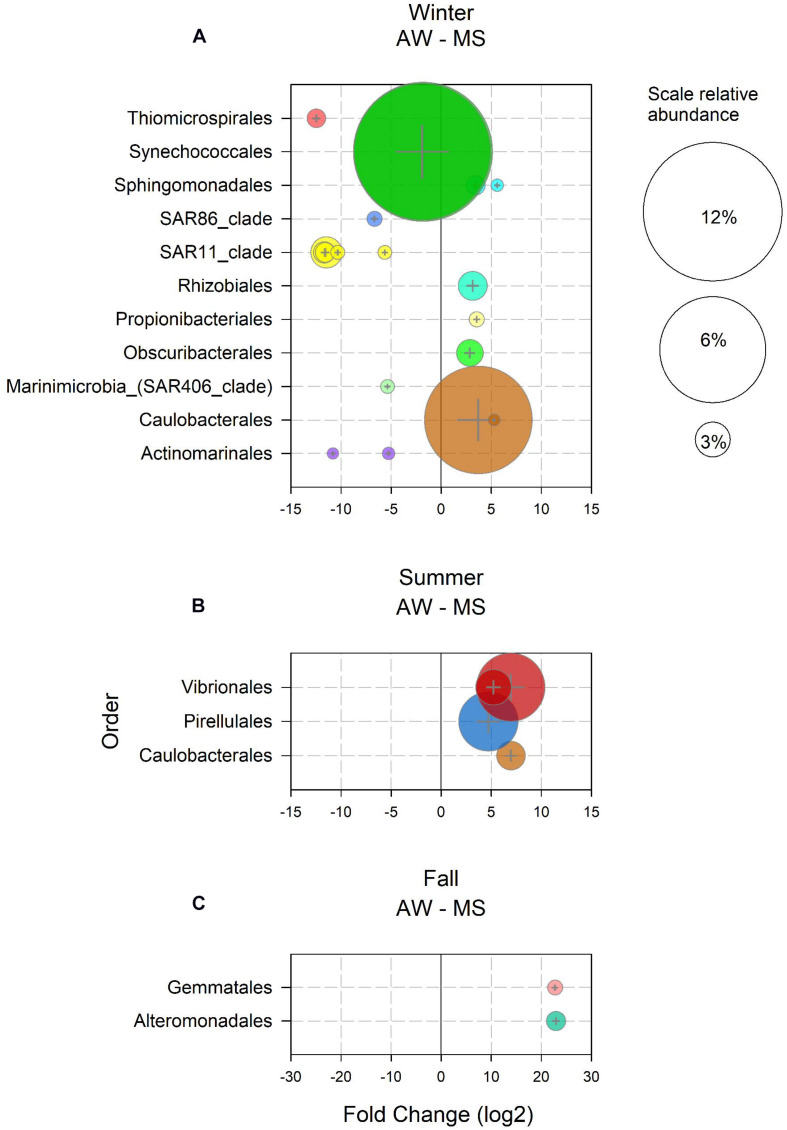
Amplicon sequence variants (ASVs) with relative abundance > 1% and a log_2_ fold change > 1 significantly (*p* < 0.05) enriched in the ambient water (AW) and in marine snow (MS) in **(A)** winter, **(B)** summer, and **(C)** fall. Bubble size represents the relative abundance and colors indicate the assigned taxonomy at the order level.

In winter, 589 ASV were enriched in the AW as compared to MS, and 19 ASVs in the MS as compared to the AW. Enriched ASVs in the AW represented 91% of the total AW community in winter, enriched ASVs in MS accounted for 26% of the total MS community. Twelve of the 589 AW-enriched ASVs had a mean abundance > 1% and were assigned to Synechococcales, SAR11, Thiomicrospirales, SAR86, SAR406, and Actinomarinales (Figure 2A). These taxa comprise groups typically found in the free-living community (Mestre et al., 2017; Preston et al., 2019), some consisting of many ecotypes, including ecotypes characteristic for the winter in temperate marine waters (Giovannoni, 2017; Hoarfrost et al., 2020). Seven MS-ASVs exhibited a mean relative abundance > 1% in winter (Figure 2A). These MS-ASVs were affiliated to orders including diverse phenotypes, such as Caulobacterales, capable of attachment and motility (Poindexter, 1964), Obscuribacterales, an order of non-phototrophic fermentative and motile cyanobacteria (Soo et al., 2014), the symbiotic nitrogen fixing Rhizobiales (Summers et al., 2013), pigmented photoautotrophs of the order Sphingomonadales (Siddaramappa et al., 2018), and Propionibacteriales, usually found on human skin (Barka et al., 2016) and in liver and kidney tissue of marine fish (Meron et al., 2020; Figure 2A). The high abundance of potential symbionts and pathogens in MS might be related to the influx of waste water from a fish cannery about 1.5 km away the sampling location (Paliaga et al., 2017). No ASVs were found enriched in spring AW and MS, indicating freshly generated and hence, recently populated MS, or a strong effect of environmental parameters on both AW and MS communities. The slight increase in seawater temperature and photosynthetically active radiation (Trisolino et al., 2018) triggered an increase in primary production as indicated by the increasing chlorophyll *a* concentrations (Supplementary Figure S5) favoring copiotrophic opportunistic prokaryotes such as Rhodobacterales (ASV4), Flavobacteriales (ASV5, 11 and 140) and Opitutales (ASV9; Wagner-Döbler and Biebl, 2006; Chafee et al., 2018; Jain et al., 2020). In summer, 50 ASVs were enriched in the AW as compared to MS and 68 ASVs in the MS over AW. AW-enriched ASVs had a mean relative abundance < 1% during the summer, however, together accounting for 7% of the total AW community. All MS-enriched ASVs together accounted for 31% of the total MS-associated community in summer. Noticeably, ASVs of the orders Vibrionales, Pirellulales, and Caulobacterales (Figure 2B) were prominently enriched in MS, in agreement with previous findings of members of these taxa attached to particles, particularly in coastal systems in the summer (DeLong et al., 1993; Mestre et al., 2017; Duret et al., 2019). The only two enriched ASVs in MS in fall were putatively assigned to Gemmatales and Alteromonadales, together accounting for 3% of the total MS-associated community (Figure 2C). Both taxa belong to previously reported particle-associated classes (Planctomycetacia and Gammaproteobacteria; Mestre et al., 2017). The low number of enriched ASVs in fall might be explained by similar reasons as described above for spring. Typically, in fall, water column mixing introduces nutrients from the nutrient-rich bottom waters into the nutrient depleted surface waters, thereby stimulating the microbial communities (Supplementary Figure S5; Ivancic et al., 2018).

#### Enriched Taxa in Different Seasons

Significant enrichment of ASVs in a specific season as compared to the previous and the following season (e.g., fall vs. winter and winter vs. spring) was tested in the AW and MS communities separately. In total, 964 ASVs were enriched in one season compared to the previous or the following season and 58 out of the 964 ASVs exhibited an average relative abundance > 1% (Supplementary Figure S6). The pattern of ASVs enriched in a specific season compared to the previous or following season was similar in the AW and MS (Mantel test: *r* = 0.7, *p* < 0.05). This indicates that seasonal conditions shape the taxonomic distribution of both, free-living and MS-associated communities.

**FIGURE 3:**
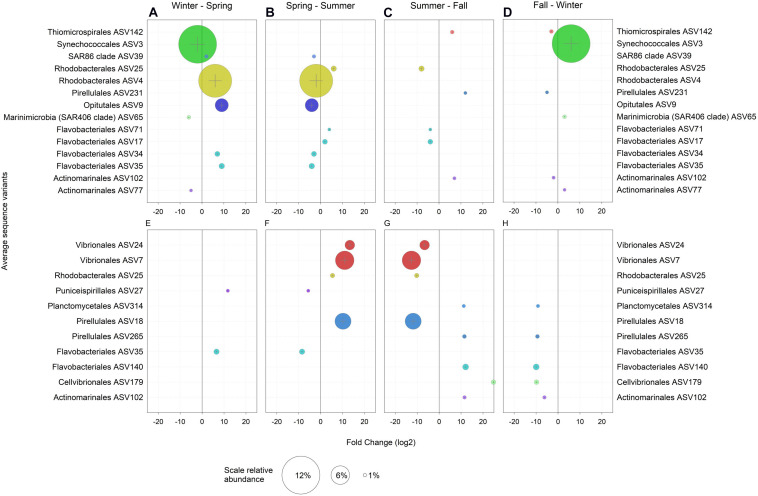
Amplicon sequence variants (ASVs) with a relative abundance > 1% and a log_2_ fold change > 1 significantly (*p* < 0.05) enriched when compared to both, the previous and the following seasons in **(A–D)** the ambient water (AW) and **(E–H)** in marine snow (MS). Bubble size represents the relative abundance and colors indicate the assigned taxonomy at the order level.

In the following, only ASVs with a relative abundance > 1% and those with significant enrichment in a particular season as compared to the previous and the following season are considered. Hence, only truly season-specific ASVs were selected (Figure 3). In the winter, the free-living community was largely characterized by one abundant *Synechococcus* ASV3, Marinimicrobia (SAR406 clade) ASV65 and Actinomarinales ASV77 (Figures 3A,D). Temperature, nutrients and light regime have been shown to structure *Synechococcus* into a large number of ecotypes, potentially including a free-living winter ecotype (Ahlgren and Rocap, 2006; Sohm et al., 2016). Marinimicrobia are ubiquitous and typically found in high abundance in the mesopelagic but also in the euphotic zone in the Adriatic Sea in winter (Yilmaz et al., 2016; Mucko et al., 2018). Actinomarinales persist throughout the year in the open Mediterranean Sea and are found below the season pycnocline during the summer (Haro-Moreno et al., 2018). Hence, the high abundance and specific occurrence of Marinimicrobia and Actinomarinales in the winter in the shallow waters of the northern Adriatic Sea are presumably due to water column mixing (Supplementary Figure S5) and lateral advection (Haro-Moreno et al., 2018).

No ASVs were specific for the winter in the MS-associated community (Figures 3E,H). However, compared to the fall, *Synechococcus* ASV2 and ASV3, both highly abundant, were enriched and dominated the MS-associated and free-living prokaryotic community in winter (Supplementary Figure S6). The high relative abundance of these two *Synechococcus* ASVs (Supplementary Figure S6) in the AW as well as in MS in winter might be due to a variety of reasons: (i) inefficient separation between MS-attached and free-living prokaryotes, particularly of abundant taxa due to sampling biases introduced by using syringes when MS is small in size; (ii) possible frequent change between the free-living and MS-associated lifestyle of *Synechococcus* cells as described for *Pseudomonas aeruginosa* (Son et al., 2015) via RTX proteins. RTX proteins are related to cell motility in cyanobacteria (Linhartová et al., 2010) and were highly abundant in winter MS (see below). (iii) Integration of copepod fecal pellets containing *Synechococcus* DNA into MS. (iv) Additionally, *Synechococcus* could initiate particle formation via release of extracellular polymeric substances (Kaltenböck and Herndl, 1992; Deng et al., 2016).

The SAR86 ASV39, Rhodobacterales ASV4, Opitutales ASV9 and two Flavobacteriales ASV34 and ASV35 were enriched in the AW in spring as compared to winter and summer (Figures 3A,B). Rhodobacterales, Flavobacteriales and Opitutae are flexible and generalist heterotrophs (Choo et al., 2007; Newton et al., 2010; Cohan, 2016; Chen et al., 2020). The SAR86 clade comprises aerobic heterotrophs with the potential of energy acquisition via proteorhodopsin (Dupont et al., 2012). Flavobacteriales ASV35 and Puniceispirillales ASV27 were also enriched in MS in spring as compared to winter and summer (Figures 3E,F). The enrichments of the Flavobacteriales ASV35 specifically in spring and of largely the same prokaryotic groups in the AW and MS in spring as compared to winter or summer (Supplementary Figure S6) indicate the similarity of AW and MS communities in spring. This suggests freshly produced MS originating from the spring phytoplankton bloom or a strong effect of environmental parameters on the prokaryotic communities in spring, as mentioned above. Typically, the increasing temperature and solar radiation in spring promote a phytoplankton bloom in the Mediterranean Sea, and hence opportunistic copiotroph prokaryotes such as *Flavobacteria* (Chafee et al., 2018) experience favorable conditions for growth.

In the summer, Flavobacteriales ASV71 and ASV17 were enriched in the AW community as compared to spring and fall (Figures 3B,C). Rhodobacterales ASV25 was enriched in the AW and MS-associated community (Figure 3). Vibrionales ASV24, ASV7 and Pirellulales ASV18 were enriched in MS and were previously reported as primarily MS-associated prokaryotes (Smith et al., 2013; Salazar et al., 2015; Steiner et al., 2019). The seasonal re-occurrence of *Vibrio* in the Adriatic and Mediterranean Sea has been linked to water stratification, elevated dissolved organic carbon concentrations and production of transparent exopolymeric particles (Zaccone et al., 2002; Tinta et al., 2015).

Similar to spring, the fall was characterized by the enrichment of the same prokaryotic groups in both the AW and MS in contrast to prokaryotic community composition in summer and winter. Actinomarinales ASV102 (Figure 3) and the abundant prokaryotic groups of the orders Rhodobacterales, Flavobacteriales, SAR11 and Pirellulales were enriched in both, the free-living and MS-associated prokaryotic community in fall (Supplementary Figure S6). Fall-specific free-living ASVs were Pirellulales ASV231 and Thiomicrospirales ASV142 (Figures 3C,D). Thiomicrospirales has been reported from oxygen depleted coastal sites and deep oxygen minimum zones linked to nitrate and the sulfur cycle (Aldunate et al., 2018; Muck et al., 2019). Pirellulales is a heterotrophic order within the class Planctomycetacia (Dedysh et al., 2020). MS-associated ASVs in fall were Planctomycetales ASV314, Cellvibrionales ASV179, Pirellulales ASV265 and Flavobacteriales ASV140 (Figures 3G,H). Planctomycetales produce secondary metabolites such as bacteriocins and ectoines (Wiegand et al., 2020) and are typically found in MS (Mestre et al., 2017; Duret et al., 2019). Cellvibrionales consist of oligotrophs as well as copiotrophs and have been reported from coastal systems and distinct nutrient-rich niches (Spring et al., 2015).

Overall, enrichment analysis revealed that ‘transition seasons,’ i.e., spring and fall, exhibited a high abundance of enriched ASVs compared to winter and summer (1,182 in total) and low abundance of habitat-specific ASVs (two in total). Whereas ‘peak seasons,’ i.e., winter and summer, showed the opposite pattern, with a comparably low number of enriched ASVs compared to spring and fall (827 in total) and a large number of habitat-specific ASVs (728 in total). High habitat specificity in AW vs. MS particularly in winter might be caused by large difference between the two habitats in terms of nutrient and possibly, oxygen availability (e.g., low oxygen microzones in MS). In the summer, MS is generally larger in size than in winter due to low turbulence conditions in the stratified water column facilitating aggregation of colloidal organic matter (Kaltenböck and Herndl, 1992; MacIntyre et al., 1996). Differences in the substrate availability between AW and MS are higher in summer and winter than in spring and fall, when phytoplankton blooms are providing labile substrate to the AW prokaryotic community (Aubry et al., 2004; Bernardi Aubry et al., 2006).

### Metagenome Analysis

#### Taxonomic Analysis

The majority of reads from the metagenomes were assigned to the domain Bacteria, ranging from 43 to 56% (Supplementary Table S3), followed by ‘not assigned’ reads (on average 41 ± 4% for all samples). Reads mapping to archaea were more abundant in winter, both in AW (3–4%) and MS (2%; Supplementary Table S3), in accordance with previous reports on seasonality and lifestyles of archaea in surface waters (Galand et al., 2010; Smith et al., 2013; Haro-Moreno et al., 2017). Eukaryotes contributed substantially less with a maximum contribution of 7–13% in MS in summer (Supplementary Table S3) in the coastal Adriatic than previously reported for MS-associated microbes in coastal (up to 44%) and deep waters (27–40% of all small subunit rRNA genes). Most reads assigned to viruses were detected in the AW in winter (3–4%) and in MS in summer (2%; Supplementary Table S3). Despite reports of lower importance of virus induced mortality as compared to grazing by protists in the Mediterranean Sea, viruses can drastically alter prokaryotic community composition, particularly in eutrophic coastal areas (Siokou-Frangou et al., 2010).

#### Free-Living and MS-Associated Eukaryotic Community Composition

**FIGURE 4:**
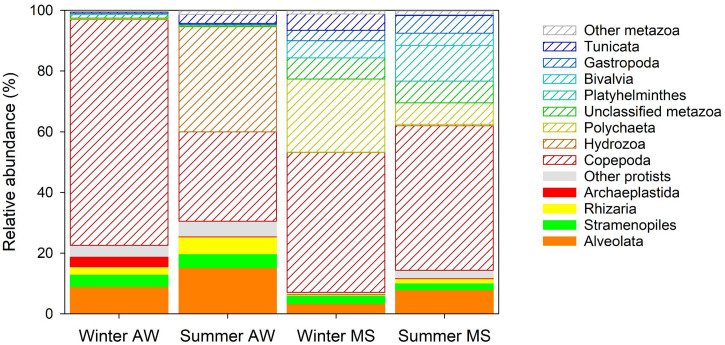
Averaged relative abundance of reads assigned to eukaryotes. The fraction of protists has color filling and the fraction of metazoa is shown as white bars with colored shading. Eukaryotic groups with lower relative abundance than 1% are grouped under “other protists” and “other metazoan.”

The largest fraction of eukaryotic reads retrieved from the metagenomes was assigned to copepods (on average 49% ± 27%, Supplementary Table S4 and Figure 4), with *Acartia clausii* (on average 15 ± 15%) as the most abundant eukaryote. Hydrozoa comprised a substantial fraction of assigned reads in AW in the summer (35 ± 6%). Certain Hydrozoa reproduce during the summer period, reaching high abundances in the Mediterranean Sea (Boero and Fresi, 1986; Di Camillo et al., 2012), suggesting that Hydrozoa sequences in summer originated from free-living hydrozoan larvae. Sequences assigned to Polychaeta (on average 24%) characterized the winter MS community and Platyhelminthes the summer MS community (on average 12%, Supplementary Table S4 and Figure 4). These findings support the notion that MS represents an important microenvironment for eukaryotes, particularly larvae of Polychaeta and juvenile turbellarians (Platyhelminthes; Bochdansky and Herndl, 1992a; Bochdansky et al., 2017). Larvae use MS as a food source and to enhance dispersal since a considerable fraction of MS is only slowly sinking or neutrally buoyant hence being laterally transported over large distances (Bochdansky and Herndl, 1992b). Furthermore, a high number of ‘not assigned’ metazoa (NA_Metazoa), Gastropoda, Bivalvia, and Tunicata were also present in the MS eukaryotic community (Supplementary Table S4 and Figure 4). The high diversity and larger proportion (89 ± 6%) of metazoan reads in MS compared to AW (73 ± 12%; Supplementary Tables S2, S3) suggest an important contribution of metazoans to the remineralization of marine snow in coastal systems (Bochdansky and Herndl, 1992b). Conversely, protists were relatively more abundant in the AW (27 ± 12%) than in MS (11 ± 6%). AW protists were mostly assigned to Alveolata (including dinoflagellates), Rhizaria (including radiolaria), Stramenopiles (including diatoms), and Archaeplastida (including red and green algae), all taxa commonly found in the northern Adriatic Sea (Monti et al., 2012; Piredda et al., 2016; Steiner et al., 2019).

#### Functional Assignment of Prokaryotic Communities

The prokaryotic community composition revealed by 16S rRNA amplicon sequences was similar to the prokaryotic community composition obtained by the metagenomes (Mantel test: *r* = 0.55, *p* < 0.05, *n* = 8, Supplementary Figure S3). This allows linking the taxonomic analysis to functional analysis based on metagenomes. The similarity was tested at the taxonomic level ‘order.’ In total, 146 ± 91 orders were detected in each sample. Thereof, 22–59 orders were common in the amplicon and metagenome data in each sample covering the main fraction of sequences (84–96%). This indicates that the similarity between the communities is determined by few abundant orders. Diversity patterns were similar as in the amplicon sequence analysis, however, less pronounced. Species richness in MS (252 ± 10.5) and Shannon diversity in MS in winter (2.7) were lower than in summer and AW samples. The 4,181 identified COGs comprised on average 11 ± 4.4% of all reads with functional assignment.

#### Enriched COGs in the Free-Living as Compared to MS-Associated Community

**FIGURE 5:**
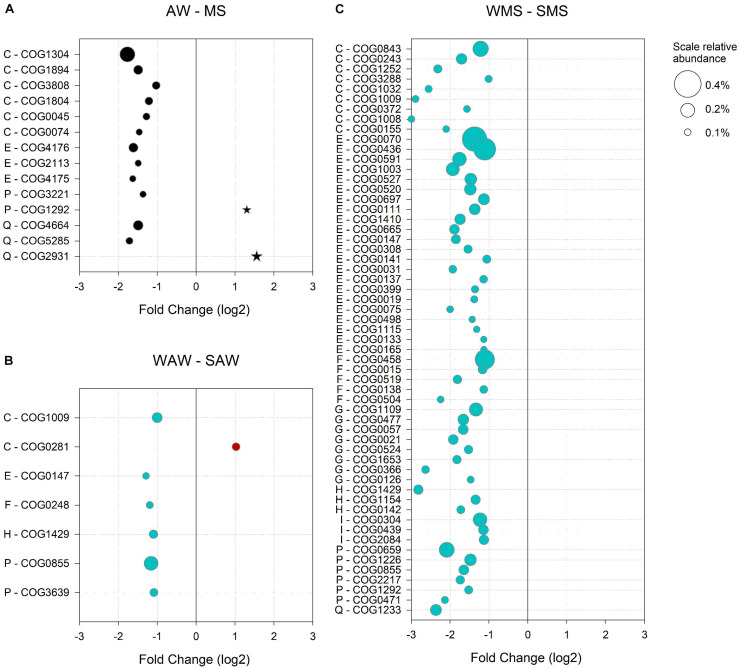
Significantly (*p* < 0.05) enriched clusters of orthologous groups (COGs) in **(A)** the ambient water (AW) versus marine snow (MS), **(B)** in winter (WAW) versus summer (SAW) ambient water communities; and **(C)** in winter (WMS) versus summer (SMS) marine snow. Only COGs with a log_2_ fold change > 1 and a relative abundance > 0.1% (indicated by bubble size) are shown. COG numbers and corresponding COG categories are indicated: E – Amino acid transport and metabolism, H – Coenzyme transport and metabolism, P – Inorganic ion transport and metabolism, C – Energy production and conversion, I – Lipid transport and metabolism, G – Carbohydrate transport and metabolism, F – Nucleotide transport and metabolism and Q – Secondary metabolites biosynthesis, transport and catabolism.

The AW community was defined by 189 significantly enriched COGs as compared to the MS-associated community, accounting for 5.2% of all COGs assigned to AW prokaryotes. A particularly high relative abundance (4%) was observed for COGs involved in metabolism, indicating that metabolic capabilities led to a differentiation of the AW from the MS-associated communities. The most abundant COGs (> 0.1%) of the supergroup ‘metabolism’ were related to ‘C – Energy production and conversion,’ including enzymes participating in terpenoid backbone biosynthesis (COG1304), precursors of carotenoids, the building blocks of the proteorhodopsin chromophore retinal (Béja et al., 2001; DeLong and Beja, 2010; Figure 5A). The latter suggests that photoheterotrophy is a survival strategy of the AW community, related to oligotrophy and carbon limitation (Moran and Miller, 2007). The vast majority of reads (65%) aligned to COGs and enriched in the AW community over MS were assigned to *Candidatus Pelagibacter* sp. HTCC7211, the alpha proteobacterium HIMB114 and *Candidatus* Puniceispirillum marinum, all abundant proteorhodopsin-containing Alphaproteobacteria (Giovannoni et al., 2005; Lee et al., 2019; Supplementary Table S5). Other genes enriched in AW communities encoded for oxidoreductases and transferases involved in the acquisition of energy from organic compounds via the citric acid cycle (COG1894, COG0074, and COG0045; Figure 5A). An acyl-CoA transferase (COG1804) involved in the catabolism of compatible solutes, such as carnitine, a betaine structurally similar to dimethylsulfoniopropionate (DMSP; Elssner et al., 2001; Todd et al., 2007; Curson et al., 2011) was abundant as well (Figure 5A). Compatible solutes are either generated by or imported into bacterial cells. Three of the most abundant AW specific COGs (category ‘E - Amino acid transport and metabolism’) were components of the ATP-binding cassette (ABC)-type proline/glycine betaine transport system (COG4176, COG2113 and COG4175), indicating potential utilization of compatible solutes as substrate or adaptations to salt stress (Figure 5A). A phytanoyl-CoA dioxygenase (COG5285) involved in the biosynthesis of polyketides (mitomycin antibiotics/fumonisin) indicated the production of antibiotics by the AW prokaryotes, possibly as a survival strategy (Figure 5A; Karimi et al., 2019).

#### Enriched COGs in the MS-Associated as Compared to the Free-Living Community

The MS-associated community was characterized by 219 enriched COGs, accounting for 2.9% of all COGs assigned to MS-associated prokaryotes. Half of the MS-enriched COGs (1.5%) were assigned to functions involved in metabolism. Particularly, a choline-glycine betaine transporter (COG1292) and a Ca^2+^ binding protein RTX toxin-related domain (COG2931) were abundant (Figure 5A). RTX proteins are involved in cell defense acting as cytotoxins in *Vibrio* or contribute to motility in *Cyanobacteria* (Linhartová et al., 2010). Furthermore, RTX proteins may form bacterial surface layers as protection and have been observed in pathogenic and non-pathogenic heterotrophic bacteria and cyanobacteria (Linhartová et al., 2010). Most reads (55%) aligned to COGs enriched in MS were assigned to diverse *Synechococcus* species (CC9311, CC9902, BL107, and WH 7805) and *Vibrio caribbeanicus* (Supplementary Table S6).

#### Seasonally Enriched COGs in the Free-Living Community

The functional potential of the AW prokaryotic community in the summer as compared to winter was characterized by 396 enriched COGs constituting 4% of the total summer COGs of the AW prokaryotes, of which approximately half (2.3%) were related to metabolism. The most abundant gene encoded the malic enzyme (COG0281), important in various metabolic pathways including the citric acid cycle (Figure 5B; Takahashi-Íñiguez et al., 2016). The main fraction of all reads aligned to enriched COGs in the summer was assigned to Pelagibacterales (53%) and unclassified Alphaproteobacteria (24%; Supplementary Table S7).

In the AW prokaryotes in winter, 531 COGs (accounting for 8.2% of COGs of the AW community) were enriched compared to the summer, and were mainly associated to Synechococcales (59%) and Pelagibacterales (24%; Supplementary Table S8). More than half (5.9%) of all winter-specific COGs were related to metabolism. Specific functions were assigned to oxidative phosphorylation (COG1009), the biosynthesis of amino acids and other secondary metabolites (COG0147), a cobalamin biosynthesis protein (COG1429) involved in porphyrin and chlorophyll metabolism, an exopolyphosphatase (COG0248), a polyphosphate kinase (COG0855) and a phosphate/phosphonate transport system (COG3639) indicating phosphorus stress (Figure 5B).

#### Seasonally Enriched COGs in the MS-Associated Community

The MS-associated community showed pronounced differences in the relative abundance of enriched COGs when comparing summer and winter. Enriched COGs in summer represented 2% and in winter 47.3%. However, a similar number of enriched COGs (853 in summer and 806 in winter) were found in both seasons. The reads aligned to summer-specific COGs were mainly assigned to Vibrionales (28%), Pelagibacterales (22%) and Rhodobacterales (13%), and mostly related to metabolism (1.2% of all enriched summer MS COGs, Supplementary Table S9). However, no single COG contributed more than 0.1% to the total COGs in MS in the summer. In winter, almost all enriched COGs were assigned to Synechococcales (92%), reflecting the main difference in the taxonomic assignment of COGs between summer and winter MS-associated communities (compare Supplementary Tables S9, S10). The most abundant COGs (> 0.1%) of the supergroup ‘metabolism’ (57 COGs) spanned eight COG categories (Figure 5C). Of particularly high relative abundance were COGs involved in nitrogen assimilation and storage (COG0436, COG0070 and COG0458), pointing to an adaptation to changing nitrogen availability (Eisenberg et al., 2000; Zhang et al., 2018). Fatty acid biosynthesis genes (COG0304 and COG0439) were enriched in the winter MS-associated community, particularly in Synechococcales members (Supplementary Table S11). Unsaturated membrane lipids in cyanobacteria protect the photosynthetic machinery from photoinhibition associated with low temperatures (Nishida and Murata, 1996). A high relative abundance of enzymes involved in photosynthesis including carotenoid biosynthesis (COG1233), terpenoid backbone biosynthesis (COG1154 and COG0142) and porphyrin and chlorophyll metabolism (COG1429 and COG0155) underlines the impact of Synechococcales on the functional potential of the MS-associated community in the winter (Figure 5C).

#### Functions of Prokaryotic Communities – Transporter Analysis

Transporters provide insights into the dynamics of nutrient and organic matter uptake of microbial communities (Bergauer et al., 2018). COGs associated with substrate transport comprised 6.6% of the MS- and AW-COGs combined. ABC transporters were the majority (44.4 ± 0.6%) of transporters, in agreement with results from diverse open ocean regions (Bergauer et al., 2018). Highest dissimilarity (determined via SIMPER analysis) between summer MS, winter MS, summer AW and winter AW was reflected in COGs (ENOG4111IMY and ENOG4111FRH) involved in transport of the compatible solute ectoine in winter AW (Supplementary Table S12), which acts as an osmoprotectant and is synthesized in conditions of salinity or temperature stress (Kuhlmann et al., 2011). Another ABC transporter (COG3638) found in high abundance particularly in winter in both AW and MS is associated to phosphonate/phosphite/phosphate transport and linked to phosphorus limitation (Gebhard and Cook, 2008; Supplementary Table S12). Many ABC transporters responsible for the dissimilarity between summer and winter were associated to iron transport and mostly present in summer in both AW and MS (COG4181 and COG3127) and in MS in the summer (COG4594; Supplementary Table S12). This includes high affinity Fe^3+^-citrate siderophores and uptake systems to pirate siderophores of other microbes (Gaballa and Helmann, 2007; Banerjee et al., 2016), pointing to iron limitation and high competition for iron in MS. Other ABC transporters characteristic for the MS-associated community in summer were important in the assembly of cytochrome *bd* terminal oxidase which is responsible for micro-aerophilic respiration and is overexpressed under nitric oxide stress (Jünemann, 1997; Mason et al., 2009).

The second most abundant transporter subfamily was the major facilitator superfamily (MFS) accounting for 21 ± 0.2% averaged over all seasons and habitats (AW and MS). The MFS is the largest family of secondary transport carriers (Saier et al., 1999; Reddy et al., 2012). Unfortunately, due to poor annotation a detailed functional description was not possible. However, a strong seasonal separation was observed, with a range of COGs seasonally enriched in summer or winter independent of the habitat, and specific COGs linked to summer MS or to summer AW (Supplementary Table S13). Other abundant superfamilies were the tripartite ATP-independent periplasmic transporter (TRAP-T) family comprising 5.7 ± 0.8% of transporter COGs averaged over all seasons and habitats, and the resistance-nodulation-cell division (RND) superfamily with 4.2 ± 0.3% of transporter COGs, also averaged over all seasons and habitats. The presence of TRAP transporters implicates preferential utilization of organic compounds and an energy saving lifestyle, which is advantageous under oligotrophic conditions (Kelly and Thomas, 2001; Bergauer et al., 2018). In this study, the few TRAP transporters showed low dissimilarity between samples and were mainly associated to a high affinity transport system encoded by the dct locus (Forward et al., 1997; Supplementary Table S14). This system transports the C4-dicarboxylates malate, succinate, and fumarate (Forward et al., 1997). The RND family includes transporters for multidrug efflux in Gram-negative bacteria (Nikaido and Takatsuka, 2009). Almost all COGs identified as members of the RND family were assigned to the membrane fusion protein HlyD (Supplementary Tables S15, S16), a specific transporter for the RTX hemolytic toxin HlyA (Pimenta et al., 2005). HlyA is a pore-forming toxin released by many pathogens (including *Vibrio* spp.) into the medium or directly into the host cell (Costa et al., 2015). However, it has been suggested that HlyD is involved in sync1217 secretion, a RTX toxin domain protein that creates an outer layer membrane barrier to toxins in *Synechococcus* (Stuart et al., 2013). In this study, 66% of all reads of all seasons and habitats combined targeting COGs of RND family transporters were aligned to COGs of the HlyD protein of *Synechococcus* (Supplementary Table S16). The large variety of HlyD assigned COGs and the heterogenous distribution through seasons and habitats indicate an important role of defense mechanisms for prokaryotic communities in this coastal system.

#### Functions of Prokaryotic Communities – Marker Gene Analysis

**FIGURE 6:**
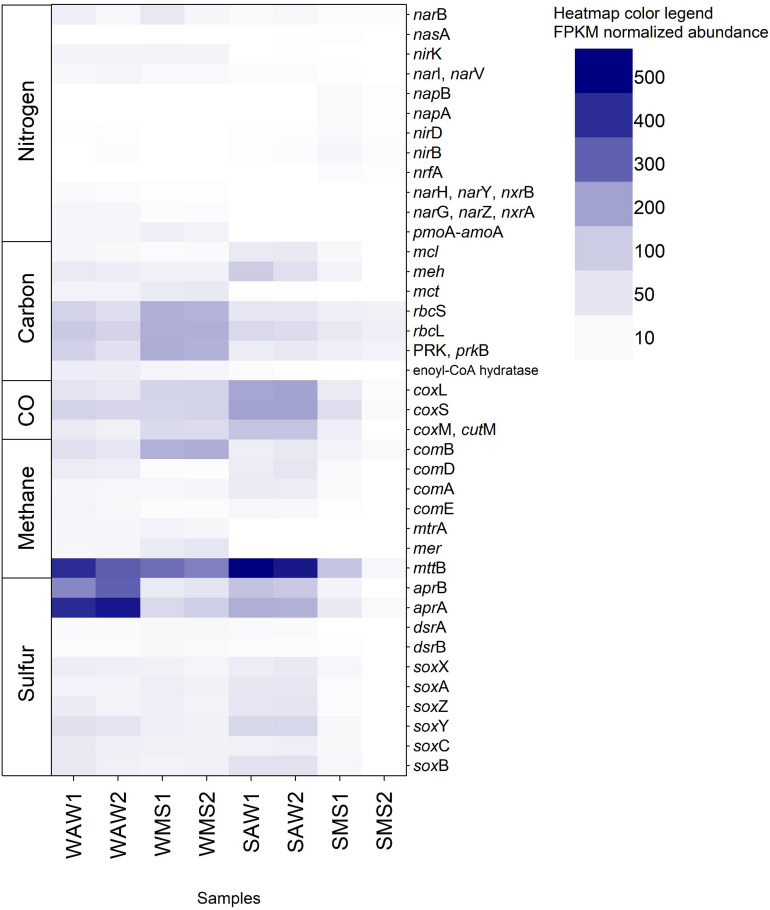
Fragments per kilo base million (FPKM) normalized abundance of the 39 KOs (Kyoto Encyclopedia of Genes and Genomes Orthology) identified and corresponding to genes involved in the carbon, nitrogen, sulfur, methane, and carbon monoxide (CO) metabolism. Gene names are listed and described in Supplementary Table S1. WAW1 and WAW2 indicate winter ambient water duplicates, SAW1 and SAW2 indicate summer ambient water duplicates, WMS1 and WMS2 correspond to winter marine snow duplicates, SMS1 and SMS2 indicate summer marine snow duplicates.

Functional prokaryotic community analysis based on 69 selected key marker genes revealed the presence of 39 KOs corresponding to diverse genes involved in carbon, nitrogen, sulfur, methane, and carbon monoxide metabolism. Genes involved in H_2_ oxidation were not present in the dataset. Interestingly, AW prokaryotes were characterized by a high relative abundance of anaerobic methanogens (*mtt*B, Figure 6) which has been typically described for marine sediments and linked to archaea (Ferry and Lessner, 2008; Sun et al., 2019). However, anaerobic methanogens have also been found in oxygenated coastal marine environments and have been hypothesized to inhabit suboxic and anoxic microenvironments within organic particles such as MS (Alldredge and Cohen, 1987; Ditchfield et al., 2012). MS collected from coastal waters as well as laboratory made artificial MS showed small patches (100 to 300 μm diameter) of reduced oxygen concentrations in marine snow (Shanks and Reeder, 1993). These microhabitats were subsequently suggested as sites of anaerobic processes (DeLong, 1992; Shanks and Reeder, 1993; Bianchi et al., 2018) including methanogenic archaea (Ditchfield et al., 2012). Our results indicate a larger potential for methanogenesis in the AW than in MS. The AW also includes small particles, which might harbor methanogens. Another possible explanation could be frequent attachment and detachment from MS by methanogenic prokaryotes, being active in microzones of MS and detaching as MS becomes dispersed (Son et al., 2015). The *apr*A and *apr*B genes were very abundant in the AW, particularly in the winter (Figure 6). The *apr*A and *apr*B genes are involved in anaerobic dissimilatory sulfate reduction by sulfate reducing organisms (SRO) such as Syntrophobacterales, Thermodesulfobacterium, Thermodesulfovibrio, *Archaeoglobus*, and some deltaproteobacterial lineages (Meyer and Kuever, 2007b). SRO have been found to be frequently associated with methanogens (Grein et al., 2013) which were abundant in winter AW as mentioned above. However, *apr* genes are also found in anoxygenic phototrophic and chemolithoautotrophic sulfur oxidizing bacteria (SOB; Frigaard and Dahl, 2008), as well as chemolithoheterotrophic SOB (Meyer and Kuever, 2007a), including the abundant Pelagibacterales (Giovannoni et al., 2005) and thus, are widespread throughout the oxygenated oceanic water column (De Corte et al., submitted).

Anaerobic metabolisms such as dissimilatory nitrate reduction (*nrf*A, *nap*A, and *nap*B) were only present in summer MS (Figure 6), indicating that large marine snow present in summer (as compared to winter as observed by SCUBA divers) was colonized by prokaryotes specialized in attachment, biofilm formation and anaerobic nitrate respiration. Most bacterial lineages can perform dissimilatory nitrite reduction to ammonium (Kuypers et al., 2018). Other genes involved in denitrification and dissimilatory nitrate reduction (*nir*K, *nar*I, and *nar*V), the assimilatory nitrate reduction gene *nar*B and genes encoding nitrate reductases – nitrite oxidoreductases (*nar*H, *nar*Y, *nxr*B, *nar*G, *nar*Z, and *nxr*A) and methane/ammonia monooxygenases (*pmo*A-*amo*A) were generally more frequent in winter than in summer in both habitats (Figure 6), although in low abundances. Nitrite and nitrate reductases are widespread among bacteria and archaea and occur in low oxygen and anoxic environments where nitrite and nitrate is present (Kuypers et al., 2018). *Nir*K is commonly used as a marker for denitrification, however, it is not exclusively found in denitrifiers (Kuypers et al., 2018). Ammonia monooxygenases occur in beta- and gammaproteobacterial classes including *Nitrosomonas*, *Nitrosococcus*, and Nitrospirae (Chain et al., 2003; Klotz et al., 2006; Daims et al., 2015) and the archaeal phylum Thaumarchaeota (Brochier-Armanet et al., 2012). None of them occurred in large abundances in this study (Supplementary Figure S3). Summer AW prokaryotes exhibited the highest relative abundance of genes involved in CO-oxidation (*cox*L, *cox*S, *cox*M, and *cut*M; Figure 6). These genes occur mostly in *Roseobacter* to supplement heterotrophic growth with inorganic carbon (Moran and Miller, 2007). In this study, however, most reads (46%) assigned to CO-oxidation COGs (COG1529, COG2080, COG1319) affiliated to the Alphaproteobacterium HIMB114 and *Candidatus* Puniceispirillum marinum (Supplementary Table S17) indicating supplementing heterotrophic growth with energy derived from carbon monoxide (Moran and Miller, 2007; Oh et al., 2010). Winter MS exhibited the highest abundance of genes from the Calvin cycle (*rbc*S, *rbc*L, and *prk*B; Figure 6) in accordance with the high abundance of cyanobacteria in MS (Supplementary Figure S3).

## Summary and Conclusion

We analyzed the community composition and functional potential of the AW and MS-associated communities over a seasonal cycle. The winter and summer were characterized by habitat specific prokaryotes, while the ‘transition seasons,’ i.e., spring and fall were defined by season-specific prokaryotes, similar in the AW and MS-associated communities.

Differences in the nutrient availability between AW and MS are larger in winter and summer than in spring and fall favoring pronounced differences in the microbial community composition in the winter and summer between AW and MS. A selective advantage of prokaryotes associated to MS possibly includes the utilization of RTX toxin-related proteins for protection and motility. Adaptations to low temperature in winter might involve protection of the photosynthetic machinery from photoinhibition by low temperature. *Synechococcus* was not significantly enriched in MS, however, most of the enriched potential functions were assigned to *Synechococcus* in MS in the winter. The prominent appearance of *Synechococcus* associated to MS suggests that MS represents a hotspot for autotrophy in winter. In contrast, the development of low oxygen microzones within MS during water column stratification and low turbulence conditions in summer offers suitable conditions for anaerobic metabolism such as nitrite respiration.

Adaptations to a free-living life-style in winter included coping with phosphorus limitation and energy acquisition from labile organic matter such as compatible solutes. In the summer, prokaryotes supplement heterotrophic growth with solar radiation energy harvested with proteorhodopsins (Gómez-Consarnau et al., 2019) or with CO-oxidation (Moran and Miller, 2007).

The ‘transition seasons’ spring and fall were largely characterized by similar composition and functional potential of the AW and MS-associated prokaryotic communities in contrast to winter and summer. The fact that the same ASVs were enriched in the AW and MS indicates that environmental parameters of the ‘transition seasons’ influence both, the AW and MS-associated communities in the same way leading to an overall convergence of the microbial community composition and functional potential between AW and MS during these ‘transition seasons.’

Overall, a pronounced seasonality in both, the AW and the MS-associated communities was detectable. The MS-associated microbial community, however, was less sensitive to seasonal changes than the AW microbial community. Thus, the MS-associated community was more stable over the seasonal cycle than the AW microbial community. The large diversity and proportion of metazoans associated with MS might substantially affect the substrate composition of marine snow and thus, prokaryotic community composition and function, particularly in the summer. In contrast, the relatively low abundance of protists (including eukaryotic phytoplankton) together with the high abundance of *Synechococcus* in winter MS suggests a major prokaryotic contribution to marine snow in this season.

## Data Availability Statement

The datasets generated in this study can be found in online repositories. The names of the repository/repositories and accession number(s) can be found below: https://www.ebi.ac.uk/ena, PRJEB38662.

## Author Contributions

PS, JG, TR, ES, and GH conceived the research. PS conducted DNA extraction and prepared 16SrRNA and metagenome sequencing. JG, AO, and PS analyzed the data under supervision and following the advices of TR and EF. PS wrote the manuscript. All authors revised the draft version and approved the final version.

## Conflict of Interest

The authors declare that the research was conducted in the absence of any commercial or financial relationships that could be construed as a potential conflict of interest.

## Footnotes

1 https://www.ebi.ac.uk/ena/data/view/PRJEB38662

2 v.3.6.2, https://www.R-project.org/

## Supplementary Material

Supplementary Figure 1 Rarefaction curves of amplicon sequences normalized to the sample with smallest number of sequences (08 February 2016 marine snow).

Supplementary Figure 2 Shannon diversity index **(A)** and species richness (number of taxa; **B)** of rarified prokaryotic communities in different sampling days based 16S rRNA amplicon sequence variant (ASV) analysis. Symbols indicate ambient water (AW) and marine snow (MS) habitats.

Supplementary Figure 3 Contribution of the 30 most abundant **(A)** MS-associated (MS) and **(B)** ambient water (AW) prokaryotic orders to the communities based on variance-stabilized 16S rRNA amplicon sequences and the 20 most abundant **(C)** MS-associated (MS) and **(D)** AW prokaryotic orders based on variance stabilized 16S rRNA sequences extracted from the metagenomes. Less abundant prokaryotic orders are grouped under “Others.” Samples from April to November were taken in 2015 and samples from February to July were taken in 2016.

Supplementary Figure 4 Venn diagrams showing the number of prokaryotic classes occurring in the ambient water (AW; blue), the marine snow (MS; yellow) and in both (overlapping circles) each season.

Supplementary Figure 5 Temperature, salinity and chlorophyll *a* profiles at station RV001 in **(A)** spring (14/04/15 black bar and 06/04/16 grey bar), **(B)** summer (26/06/15 black bar and 19/07/16 gray bar), **(C)** fall (26/10/15 black bar and 09/11/15 gray bar) and **(D)** winter (20/01/16).

Supplementary Figure 6 Amplicon sequence variants (ASVs) with a relative abundance > 1% and a log_2_ fold change > 1 significantly (*p* < 0.05) enriched in one of two consecutive seasons in **(A)** the ambient water and **(B)** in marine snow (MS). Bubble size represents the relative abundance and colors indicate the assigned taxonomy at the order level.

Supplementary Table 1 Date, sample type and diversity indices of samples rarified to the lowest number of amplicon sequence variants (ASVs).

Supplementary Table 2 SIMPER analysis based on relative abundance of reads assigned to eukaryotes. WAW, winter ambient water; SAW, summer ambient water; WMS, winter marine snow; SMS, summer marine snow.

Supplementary Table 3 Abundance and taxonomic assignment of trimmed reads, and diversity indexes of the metagenomes.

Supplementary Table 4 Taxonomy, number of reads and relative abundance of reads aligned to significantly enriched species in the ambient water (AW).

Supplementary Table 5 Taxonomy, number of reads and relative abundance of reads aligned to significantly enriched species in marine snow (MS).

Supplementary Table 6 Taxonomy, number of reads and relative abundance of reads aligned to significantly enriched species in summer ambient water (AW).

Supplementary Table 7 Taxonomy, number of reads and relative abundance of reads aligned to significantly enriched species in winter ambient water (AW).

Supplementary Table 8 Taxonomy, number of reads and relative abundance of reads aligned to significantly enriched species in summer marine snow (MS).

Supplementary Table 9 Taxonomy, number of reads and relative abundance of reads aligned to significantly enriched species in winter marine snow (MS).

Supplementary Table 10 Taxonomy, number of reads and relative abundance of reads aligned to COG0304 and COG0439 in winter marine snow (MS).

Supplementary Table 11 SIMPER analysis based on variance stabilized abundance of reads aligned to ATP-binding cassette (ABC) transporter clusters of orthologous groups (COGs). WAW, winter ambient water; SAW, summer ambient water; WMS, winter marine snow; SMS, summer marine snow.

Supplementary Table 12 SIMPER analysis based on variance stabilized abundance of reads aligned to major facilitator superfamily (MFS) transporter clusters of orthologous groups (COGs). WAW, winter ambient water; SAW, summer ambient water; WMS, winter marine snow; SMS, summer marine snow.

Supplementary Table 13 SIMPER analysis based on variance stabilized abundance of reads aligned to tripartite ATP-independent periplasmic transporter (TRAP-T) family clusters of orthologous groups (COGs). WAW, winter ambient water; SAW, summer ambient water; WMS, winter marine snow; SMS, summer marine snow.

Supplementary Table 14 **S**IMPER analysis based on variance stabilized abundance of reads aligned to resistance-nodulation-cell division (RND) superfamily clusters of orthologous groups (COGs). WAW, winter ambient water; SAW, summer ambient water; WMS, winter marine snow; SMS, summer marine snow.

Supplementary Table 15 List of clusters of orthologous groups (COGs) associated with the hylD gene and a table showing the taxonomic affiliation (Taxa), average (AV), sum and relative abundance (%) of reads aligned to clusters of orthologous groups (COGs) associated with the gene hylD of all samples.

Supplementary Table 16 Taxonomy (Taxa), number of reads (Reads) and relative abundance (%) of reads aligned to COGs associated to CO-oxidation (COG1529, COG2080, COG1319) in winter MS.

Supplementary Table 17 Marker genes used normalized abundance. The presence (+) in at least one sample of the dataset is indicated. WAW1 and WAW2, winter ambient water duplicates; SAW1 and SAW2, summer ambient water duplicates; WMS1 and WMS2, winter marine snow duplicates; SMS1 and SMS2, summer marine snow duplicates.

## References

[B1] Acinas S. G. Antón J. Rodríguez-Valera F. (1999). Diversity of free-living and attached bacteria in offshore western Mediterranean waters as depicted by analysis of genes encoding 16S rRNA. *Appl. Environ. Microbiol.* 65 514–522. 10.1128/aem.65.2.514-522.1999 9925576 PMC91055

[B2] Acinas S. G. Sánchez P. Salazar G. Cornejo-Castillo F. M. Sebastián M. Logares R. (2019). Metabolic architecture of the deep ocean microbiome. *bioRxiv [Preprint]* 10.1101/635680

[B3] Ahlgren N. A. Rocap G. (2006). Culture isolation and culture-independent clone libraries reveal new marine *Synechococcus* ecotypes with distinctive light and N physiologies. *Appl. Environ. Microbiol.* 72 7193–7204. 10.1128/aem.00358-06 16936060 PMC1636174

[B4] Aldunate M. De la Iglesia R. Bertagnolli A. D. Ulloa O. (2018). Oxygen modulates bacterial community composition in the coastal upwelling waters off central Chile. *Deep Sea Res. II Top. Stud. Oceanogr.* 156 68–79. 10.1016/j.dsr2.2018.02.001

[B5] Alldredge A. L. Cohen Y. (1987). Can microscale chemical patches persist in the sea? Microelectrode study of marine snow, fecal pellets. *Science* 235 689–691. 10.1126/science.235.4789.689 17833630

[B6] Alldredge A. L. Silver M. W. (1988). Characteristics, dynamics and significance of marine snow. *Prog. Oceanogr.* 20 41–82. 10.1016/0079-6611(88)90053-5

[B7] Aubry F. B. Berton A. Bastianini M. Socal G. Acri F. (2004). Phytoplankton succession in a coastal area of the NW Adriatic, over a 10-year sampling period (1990–1999). *Cont. Shelf Res.* 24 97–115. 10.1016/j.csr.2003.09.007

[B8] Banerjee S. Paul S. Nguyen L. T. Chu B. C. Vogel H. J. (2016). FecB, a periplasmic ferric-citrate transporter from *E. coli*, can bind different forms of ferric-citrate as well as a wide variety of metal-free and metal-loaded tricarboxylic acids. *Metallomics* 8 125–133. 10.1039/c5mt00218d 26600288

[B9] Barka E. A. Vatsa P. Sanchez L. Gaveau-Vaillant N. Jacquard C. Klenk H.-P. (2016). Taxonomy, physiology, and natural products of *Actinobacteria*. *Microbiol. Mol. Biol. Rev.* 80 1–43. 10.1128/mmbr.00019-15 26609051 PMC4711186

[B10] Béja O. Spudich E. N. Spudich J. L. Leclerc M. DeLong E. F. (2001). Proteorhodopsin phototrophy in the ocean. *Nature* 411 786–789. 10.1038/35081051 11459054

[B11] Bergauer K. Fernandez-Guerra A. Garcia J. A. Sprenger R. R. Stepanauskas R. Pachiadaki M. G. (2018). Organic matter processing by microbial communities throughout the Atlantic water column as revealed by metaproteomics. *Proc. Natl. Acad. Sci. U.S.A.* 115 E400–E408. 29255014 10.1073/pnas.1708779115 PMC5776962

[B12] Bernardi Aubry F. Acri F. Bastianini M. Bianchi F. Cassin D. Pugnetti A. (2006). Seasonal and interannual variations of phytoplankton in the Gulf of Venice (Northern Adriatic Sea). *Chem. Ecol.* 22 S71–S91.

[B13] Bianchi D. Weber T. S. Kiko R. Deutsch C. (2018). Global niche of marine anaerobic metabolisms expanded by particle microenvironments. *Nat. Geosci.* 11 263–268. 10.1038/s41561-018-0081-0

[B14] Bochdansky A. B. Clouse M. A. Herndl G. J. (2017). Eukaryotic microbes, principally fungi and labyrinthulomycetes, dominate biomass on bathypelagic marine snow. *ISME J.* 11 362–373. 10.1038/ismej.2016.113 27648811 PMC5270556

[B15] Bochdansky A. B. Herndl G. J. (1992a). Ecology of amorphous aggregations (marine snow) in the Northern Adriatic Sea. V. Role of fecal pellets in marine snow. *Mar. Ecol. Prog. Ser.* 89 297–297. 10.3354/meps089297

[B16] Bochdansky A. B. Herndl G. J. (1992b). Ecology of amorphous aggregations (marine snow) in the Northern Adriatic Sea. 111. Zooplankton interactions with marine snow. *Mar. Ecol. Prog. Ser.* 87 135–146. 10.3354/meps087135

[B17] Boero F. Fresi E. (1986). Zonation and evolution of a rocky bottom hydroid community. *Mar. Ecol.* 7 123–150. 10.1111/j.1439-0485.1986.tb00152.x

[B18] Bolger A. M. Lohse M. Usadel B. (2014). Trimmomatic: a flexible trimmer for Illumina sequence data. *Bioinformatics* 30 2114–2120. 10.1093/bioinformatics/btu170 24695404 PMC4103590

[B19] Brochier-Armanet C. Gribaldo S. Forterre P. (2012). Spotlight on the Thaumarchaeota. *ISME J.* 6 227–230. 10.1038/ismej.2011.145 22071344 PMC3260508

[B20] Buesseler K. O. Trull T. W. Steinberg D. K. Silver M. W. Siegel D. A. Saitoh S.-I. (2008). VERTIGO (VERtical transport in the global ocean): a study of particle sources and flux attenuation in the North Pacific. *Deep Sea Res. II Top. Stud. Oceanogr.* 55 1522–1539. 10.1016/j.dsr2.2008.04.024

[B21] Callahan B. J. McMurdie P. J. Rosen M. J. Han A. W. Johnson A. J. A. Holmes S. P. (2016). DADA2: high-resolution sample inference from Illumina amplicon data. *Nat. Methods* 13 581–583. 10.1038/nmeth.3869 27214047 PMC4927377

[B22] Chafee M. Fernàndez-Guerra A. Buttigieg P. L. Gerdts G. Eren A. M. Teeling H. (2018). Recurrent patterns of microdiversity in a temperate coastal marine environment. *ISME J.* 12 237–252. 10.1038/ismej.2017.165 29064479 PMC5739018

[B23] Chain P. Lamerdin J. Larimer F. Regala W. Lao V. Land M. (2003). Complete genome sequence of the ammonia-oxidizing bacterium and obligate chemolithoautotroph *Nitrosomonas europaea*. *J. Bacteriol.* 185 2759–2773. 10.1128/jb.185.9.2759-2773.2003 12700255 PMC154410

[B24] Chen Y.-J. Leung P. M. Bay S. K. Hugenholtz P. Kessler A. J. Shelley G. (2020). Metabolic flexibility allows generalist bacteria to become dominant in a frequently disturbed ecosystem. *bioRxiv [Preprint]* 10.1101/2020.02.12.945220 PMC8443593 33941890

[B25] Cho B. C. Azam F. (1988). Major role of bacteria in biogeochemical fluxes in the ocean’s interior. *Nature* 332 441–443. 10.1038/332441a0

[B26] Choo Y.-J. Lee K. Song J. Cho J.-C. (2007). *Puniceicoccus vermicola* gen. nov., sp. nov., a novel marine bacterium, and description of *Puniceicoccaceae* fam. nov., *Puniceicoccales* ord. nov., *Opitutaceae* fam. nov., *Opitutales* ord. nov. and *Opitutae* classis nov. in the phylum ‘*Verrucomicrobia*’. *Int. J. Syst. Evol. Microbiol.* 57 532–537. 10.1099/ijs.0.64616-0 17329779

[B27] Chow C.-E. T. Sachdeva R. Cram J. A. Steele J. A. Needham D. M. Patel A. (2013). Temporal variability and coherence of euphotic zone bacterial communities over a decade in the Southern California Bight. *ISME J.* 7 2259–2273. 10.1038/ismej.2013.122 23864126 PMC3834854

[B28] Cohan F. M. (2016). Bacterial speciation: genetic sweeps in bacterial species. *Curr. Biol.* 26 R112–R115. 26859266 10.1016/j.cub.2015.10.022

[B29] Costa T. R. Felisberto-Rodrigues C. Meir A. Prevost M. S. Redzej A. Trokter M. (2015). Secretion systems in Gram-negative bacteria: structural and mechanistic insights. *Nat. Rev. Microbiol.* 13 343–359. 10.1038/nrmicro3456 25978706

[B30] Cozzi S. Ivanèić I. Catalano G. Djakovac T. Degobbis D. (2004). Dynamics of the oceanographic properties during mucilage appearance in the Northern Adriatic Sea: analysis of the 1997 event in comparison to earlier events. *J. Mar. Syst.* 50 223–241. 10.1016/j.jmarsys.2004.01.007

[B31] Curson A. R. Todd J. D. Sullivan M. J. Johnston A. W. (2011). Catabolism of dimethylsulphoniopropionate: microorganisms, enzymes and genes. *Nat. Rev. Microbiol.* 9 849–859. 10.1038/nrmicro2653 21986900

[B32] Daims H. Lebedeva E. V. Pjevac P. Han P. Herbold C. Albertsen M. (2015). Complete nitrification by *Nitrospira* bacteria. *Nature* 528 504–509. 10.1038/nature16461 26610024 PMC5152751

[B33] De La Rocha C. L. Passow U. (2007). Factors influencing the sinking of POC and the efficiency of the biological carbon pump. *Deep Sea Res. II Top. Stud. Oceanogr.* 54 639–658. 10.1016/j.dsr2.2007.01.004

[B34] Dedysh S. N. Kulichevskaya I. S. Beletsky A. V. Ivanova A. A. Rijpstra W. I. C. Damsté J. S. S. (2020). *Lacipirellula parvula* gen. nov., sp. nov., representing a lineage of planctomycetes widespread in low-oxygen habitats, description of the family *Lacipirellulaceae* fam. nov. and proposal of the orders *Pirellulales* ord. nov., *Gemmatales* ord. nov. and *Isosphaerales* ord. nov. *Syst. Appl. Microbiol.* 43:126050. 10.1016/j.syapm.2019.126050 31882205 PMC6995999

[B35] DeLong E. F. (1992). Archaea in coastal marine environments. *Proc. Natl. Acad. Sci. U.S.A.* 89 5685–5689. 1608980 10.1073/pnas.89.12.5685 PMC49357

[B36] DeLong E. F. Beja O. (2010). The light-driven proton pump proteorhodopsin enhances bacterial survival during tough times. *PLoS Biol.* 8:e1000359. 10.1371/journal.pbio.1000359 20436957 PMC2860490

[B37] DeLong E. F. Franks D. G. Alldredge A. L. (1993). Phylogenetic diversity of aggregate-attached vs. free-living marine bacterial assemblages. *Limnol. Oceanogr.* 38 924–934. 10.4319/lo.1993.38.5.0924

[B38] Deng W. Cruz B. N. Neuer S. (2016). Effects of nutrient limitation on cell growth, TEP production and aggregate formation of marine *Synechococcus*. *Aquat. Microb. Ecol.* 78 39–49. 10.3354/ame01803

[B39] Di Camillo C. Betti F. Bo M. Martinelli M. Puce S. Vasapollo C. (2012). Population dynamics of *Eudendrium racemosum* (Cnidaria, Hydrozoa) from the north Adriatic Sea. *Mar. Biol.* 159 1593–1609. 10.1007/s00227-012-1948-z

[B40] Diepenbroek M. Glöckner F. O. Grobe P. Güntsch A. Huber R. König-Ries B. (2014). “Towards an integrated biodiversity and ecological research data management and archiving platform: the German federation for the curation of biological data (GFBio),” in *Informatik 2014*, eds Plödereder E. Grunske L. Schneider E. Ull D., (Bonn: Gesellschaft für Informatik).

[B41] Ditchfield A. K. Wilson S. T. Hart M. C. Purdy K. J. Green D. H. Hatton A. D. (2012). Identification of putative methylotrophic and hydrogenotrophic methanogens within sedimenting material and copepod faecal pellets. *Aquat. Microb. Ecol.* 67 151–160. 10.3354/ame01585

[B42] Ducklow H. W. Steinberg D. K. Buesseler K. O. (2001). Upper ocean carbon export and the biological pump. *Oceanography* 14 50–58. 10.5670/oceanog.2001.06

[B43] Dupont C. L. Rusch D. B. Yooseph S. Lombardo M.-J. Richter R. A. Valas R. (2012). Genomic insights to SAR86, an abundant and uncultivated marine bacterial lineage. *ISME J.* 6 1186–1199. 10.1038/ismej.2011.189 22170421 PMC3358033

[B44] Duret M. T. Lampitt R. S. Lam P. (2019). Prokaryotic niche partitioning between suspended and sinking marine particles. *Environ. Microbiol. Rep.* 11 386–400. 10.1111/1758-2229.12692 30246414

[B45] Eisenberg D. Gill H. S. Pfluegl G. M. Rotstein S. H. (2000). Structure–function relationships of glutamine synthetases. *Biochim. Biophys. Acta* 1477 122–145. 10.1016/s0167-4838(99)00270-8 10708854

[B46] Elssner T. Engemann C. Baumgart K. Kleber H.-P. (2001). Involvement of coenzyme A esters and two new enzymes, an enoyl-CoA hydratase and a CoA-transferase, in the hydration of crotonobetaine to L-carnitine by *Escherichia coli*. *Biochemistry* 40 11140–11148. 10.1021/bi0108812 11551212

[B47] Eren A. M. Maignien L. Sul W. J. Murphy L. G. Grim S. L. Morrison H. G. (2013). Oligotyping: differentiating between closely related microbial taxa using 16S rRNA gene data. *Methods Ecol. Evol.* 4 1111–1119. 10.1111/2041-210x.12114 24358444 PMC3864673

[B48] Ferry J. G. Lessner D. J. (2008). Methanogenesis in marine sediments. *Ann. N. Y. Acad. Sci.* 1125 147–157. 10.1196/annals.1419.007 18378593

[B49] Forward J. A. Behrendt M. C. Wyborn N. R. Cross R. Kelly D. J. (1997). TRAP transporters: a new family of periplasmic solute transport systems encoded by the dctPQM genes of *Rhodobacter capsulatus* and by homologs in diverse gram-negative bacteria. *J. Bacteriol.* 179 5482–5493. 10.1128/jb.179.17.5482-5493.1997 9287004 PMC179420

[B50] Frigaard N.-U. Dahl C. (2008). Sulfur metabolism in phototrophic sulfur bacteria. *Adv. Microb. Physiol.* 54 103–200. 10.1016/s0065-2911(08)00002-7 18929068

[B51] Fuhrman J. A. Cram J. A. Needham D. M. (2015). Marine microbial community dynamics and their ecological interpretation. *Nat. Rev. Microbiol.* 13 133–146. 10.1038/nrmicro3417 25659323

[B52] Gaballa A. Helmann J. D. (2007). Substrate induction of siderophore transport in *Bacillus subtilis* mediated by a novel one-component regulator. *Mol. Microbiol.* 66 164–173. 10.1111/j.1365-2958.2007.05905.x 17725565 PMC3022416

[B53] Galand P. E. Gutiérrez-Provecho C. Massana R. Gasol J. M. Casamayor E. O. (2010). Inter-annual recurrence of archaeal assemblages in the coastal NW Mediterranean Sea (Blanes Bay Microbial Observatory). *Limnol. Oceanogr.* 55 2117–2125. 10.4319/lo.2010.55.5.2117

[B54] Gebhard S. Cook G. M. (2008). Differential regulation of high-affinity phosphate transport systems of *Mycobacterium smegmatis*: identification of PhnF, a repressor of the phnDCE operon. *J. Bacteriol.* 190 1335–1343. 10.1128/jb.01764-07 18083811 PMC2238217

[B55] Giovannoni S. J. (2017). SAR11 bacteria: the most abundant plankton in the oceans. *Annu. Rev. Mar. Sci.* 9 231–255. 10.1146/annurev-marine-010814-015934 27687974

[B56] Giovannoni S. J. Tripp H. J. Givan S. Podar M. Vergin K. L. Baptista D. (2005). Genome streamlining in a cosmopolitan oceanic bacterium. *Science* 309 1242–1245. 10.1126/science.1114057 16109880

[B57] Gómez-Consarnau L. Raven J. A. Levine N. M. Cutter L. S. Wang D. Seegers B. (2019). Microbial rhodopsins are major contributors to the solar energy captured in the sea. *Sci. Adv.* 5:eaaw8855. 10.1126/sciadv.aaw8855 31457093 PMC6685716

[B58] Graham E. D. Tully B. J. (2020). Marine Dadabacteria exhibit genome streamlining and phototrophy-driven niche partitioning. *bioRxiv [Preprint]* 10.1101/2020.06.22.165886 PMC8115339 33230264

[B59] Grein F. Ramos A. R. Venceslau S. S. Pereira I. A. (2013). Unifying concepts in anaerobic respiration: insights from dissimilatory sulfur metabolism. *Biochim. Biophys. Acta* 1827 145–160. 10.1016/j.bbabio.2012.09.001 22982583

[B60] Gruber-Vodicka H. R. Seah B. K. Pruesse E. (2019). phyloFlash—rapid SSU rRNA profiling and targeted assembly from metagenomes. *bioRxiv [Preprint]* 10.1101/521922 PMC7593591 33109753

[B61] Hammer Ø. Harper D. Ryan P. (2001). *PAST-Palaeontological Statistics.* Available at: www.uv.es/pardomv/pe/2001_1/past/pastprog/past.pdf (accessed September 25, 2009).

[B62] Haro-Moreno J. M. López-Pérez M. José R. Picazo A. Camacho A. Rodriguez-Valera F. (2018). Fine metagenomic profile of the Mediterranean stratified and mixed water columns revealed by assembly and recruitment. *Microbiome* 6:128. 10.1186/s40168-018-0513-5 PMC6040077 29991350

[B63] Haro-Moreno J. M. Rodriguez-Valera F. López-García P. Moreira D. Martin-Cuadrado A.-B. (2017). New insights into marine group III Euryarchaeota, from dark to light. *ISME J.* 11 1102–1117. 10.1038/ismej.2016.188 28085158 PMC5437922

[B64] Herndl G. J. (1992). Marine snow in the Northern Adriatic Sea: possible causes and consequences for a shallow ecosystem. *Aquat. Microb. Ecol.* 6 149–172.

[B65] Herndl G. J. Peduzzi P. (1988). The ecology of amorphous aggregations (Marine Snow) in the Northern Adriatic Sea. *Mar. Ecol.* 9 79–90. 10.1111/j.1439-0485.1988.tb00199.x

[B66] Herndl G. J. Reinthaler T. (2013). Microbial control of the dark end of the biological pump. *Nat. Geosci.* 6 718–724. 10.1038/ngeo1921 24707320 PMC3972885

[B67] Herrmann M. Wegner C.-E. Taubert M. Geesink P. Lehmann K. Yan L. (2019). Predominance of *Cand*. Patescibacteria in groundwater is caused by their preferential mobilization from soils and flourishing under oligotrophic conditions. *Front. Microbiol.* 10:1407. 10.3389/fmicb.2019.01407 31281301 PMC6596338

[B68] Hoarfrost A. Nayfach S. Ladau J. Yooseph S. Arnosti C. Dupont C. L. (2020). Global ecotypes in the ubiquitous marine clade SAR86. *ISME J.* 14 178–188. 10.1038/s41396-019-0516-7 31611653 PMC6908720

[B69] Hugoni M. Taib N. Debroas D. Domaizon I. Dufournel I. J. Bronner G. (2013). Structure of the rare archaeal biosphere and seasonal dynamics of active ecotypes in surface coastal waters. *Proc. Natl. Acad. Sci. U.S.A.* 110 6004–6009. 10.1073/pnas.1216863110 23536290 PMC3625260

[B70] Huson D. H. Auch A. F. Qi J. Schuster S. C. (2007). MEGAN analysis of metagenomic data. *Genome Res.* 17 377–386. 10.1101/gr.5969107 17255551 PMC1800929

[B71] Hyatt D. LoCascio P. F. Hauser L. J. Uberbacher E. C. (2012). Gene and translation initiation site prediction in metagenomic sequences. *Bioinformatics* 28 2223–2230. 10.1093/bioinformatics/bts429 22796954

[B72] Ivancic I. Paliaga P. Pfannkuchen M. Djakovac T. Najdek M. Steiner P. (2018). Seasonal variations in extracellular enzymatic activity in marine snow-associated microbial communities and their impact on the surrounding water. *FEMS Microbiol. Ecol.* 94:fiy198. 10.1093/femsec/fiy198 30299466

[B73] Jain A. Krishnan K. P. Begum N. Singh A. Thomas F. A. Gopinath A. (2020). Response of bacterial communities from Kongsfjorden (Svalbard, Arctic Ocean) to macroalgal polysaccharide amendments. *Mar. Environ. Res.* 155:104874. 10.1016/j.marenvres.2020.104874 31975691

[B74] Jünemann S. (1997). Cytochrome bd terminal oxidase. *Biochim. Biophys. Acta* 1321 107–127. 9332500 10.1016/s0005-2728(97)00046-7

[B75] Kaltenböck E. Herndl G. J. (1992). Ecology of amorphous aggregations (marine snow) in the Northern Adriatic Sea. IV. Dissolved nutrients and the autotrophic community associated with marine snow. *Mar. Ecol. Prog. Ser.* 87 147–159. 10.3354/meps087147

[B76] Karimi E. Keller-Costa T. Slaby B. M. Cox C. J. da Rocha U. N. Hentschel U. (2019). Genomic blueprints of sponge-prokaryote symbiosis are shared by low abundant and cultivatable *Alphaproteobacteria*. *Sci. Rep.* 9:1999. 10.1038/s41598-019-38737-x PMC6374434 30760820

[B77] Kelly D. J. Thomas G. H. (2001). The tripartite ATP-independent periplasmic (TRAP) transporters of bacteria and archaea. *FEMS Microbiol. Rev.* 25 405–424. 10.1111/j.1574-6976.2001.tb00584.x 11524131

[B78] Klotz M. G. Arp D. J. Chain P. S. El-Sheikh A. F. Hauser L. J. Hommes N. G. (2006). Complete genome sequence of the marine, chemolithoautotrophic, ammonia-oxidizing bacterium *Nitrosococcus oceani* ATCC 19707. *Appl. Environ. Microbiol.* 72 6299–6315. 10.1128/aem.00463-06 16957257 PMC1563620

[B79] Kuhlmann A. U. Hoffmann T. Bursy J. Jebbar M. Bremer E. (2011). Ectoine and hydroxyectoine as protectants against osmotic and cold stress: uptake through the SigB-controlled betaine-choline-carnitine transporter-type carrier EctT from *Virgibacillus pantothenticus*. *J. Bacteriol.* 193 4699–4708. 10.1128/jb.05270-11 21764932 PMC3165649

[B80] Kuypers M. M. Marchant H. K. Kartal B. (2018). The microbial nitrogen-cycling network. *Nat. Rev. Microbiol.* 16 263–276. 10.1038/nrmicro.2018.9 29398704

[B81] Lee J. Kwon K. K. Lim S.-I. Song J. Choi A. R. Yang S.-H. (2019). Isolation, cultivation, and genome analysis of proteorhodopsin-containing SAR116-clade strain *Candidatus* Puniceispirillum marinum IMCC1322. *J. Microbiol.* 57 676–687. 10.1007/s12275-019-9001-2 31201724

[B82] Li D. Liu C.-M. Luo R. Sadakane K. Lam T.-W. (2015). MEGAHIT: an ultra-fast single-node solution for large and complex metagenomics assembly via succinct de *Bruijn* graph. *Bioinformatics* 31 1674–1676. 10.1093/bioinformatics/btv033 25609793

[B83] Linhartová I. Bumba L. Mašín J. Basler M. Osička R. Kamanová J. (2010). RTX proteins: a highly diverse family secreted by a common mechanism. *FEMS Microbiol. Rev.* 34 1076–1112. 10.1111/j.1574-6976.2010.00231.x 20528947 PMC3034196

[B84] Love M. I. Huber W. Anders S. (2014). Moderated estimation of fold change and dispersion for RNA-seq data with DESeq2. *Genome Biol.* 15:550. 10.1186/s13059-014-0550-8 PMC4302049 25516281

[B85] MacIntyre S. Alldredge A. Gotschalk C. (1996). Accumulation of marine snow at density discontinuities in the water column. *Oceanogr. Lit. Rev.* 3:252.

[B86] Mason M. G. Shepherd M. Nicholls P. Dobbin P. S. Dodsworth K. S. Poole R. K. (2009). Cytochrome bd confers nitric oxide resistance to *Escherichia coli*. *Nat. Chem. Biol.* 5 94–96. 10.1038/nchembio.135 19109594

[B87] McMurdie P. J. Holmes S. (2013). phyloseq: an R package for reproducible interactive analysis and graphics of microbiome census data. *PLoS One* 8:e61217. 10.1371/journal.pone.0061217 23630581 PMC3632530

[B88] McMurdie P. J. Holmes S. (2014). Waste not, want not: why rarefying microbiome data is inadmissible. *PLoS Comput. Biol.* 10:e1003531. 10.1371/journal.pcbi.1003531 24699258 PMC3974642

[B89] Meron D. Davidovich N. Ofek-Lalzar M. Berzak R. Scheinin A. Regev Y. (2020). Specific pathogens and microbial abundance within liver and kidney tissues of wild marine fish from the Eastern Mediterranean Sea. *Microb. Biotechnol.* 13 770–780. 10.1111/1751-7915.13537 32059079 PMC7111072

[B90] Mestre M. Borrull E. Sala M. Gasol J. M. (2017). Patterns of bacterial diversity in the marine planktonic particulate matter continuum. *ISME J.* 11 999–1010. 10.1038/ismej.2016.166 28045454 PMC5364349

[B91] Meyer B. Kuever J. (2007a). Molecular analysis of the diversity of sulfate-reducing and sulfur-oxidizing prokaryotes in the environment, using aprA as functional marker gene. *Appl. Environ. Microbiol.* 73 7664–7679. 10.1128/aem.01272-07 17921272 PMC2168068

[B92] Meyer B. Kuever J. (2007b). Phylogeny of the alpha and beta subunits of the dissimilatory adenosine-5′-phosphosulfate (APS) reductase from sulfate-reducing prokaryotes–origin and evolution of the dissimilatory sulfate-reduction pathway. *Microbiology* 153 2026–2044. 10.1099/mic.0.2006/003152-0 17600048

[B93] Monti M. Minocci M. Milani L. Umani S. F. (2012). Seasonal and interannual dynamics of microzooplankton abundances in the Gulf of Trieste (Northern Adriatic Sea, Italy). *Estuar. Coast. Shelf Sci.* 115 149–157. 10.1016/j.ecss.2012.03.032

[B94] Moran M. A. Miller W. L. (2007). Resourceful heterotrophs make the most of light in the coastal ocean. *Nat. Rev. Microbiol.* 5 792–800. 10.1038/nrmicro1746 17828280

[B95] Muck S. De Corte D. Clifford E. L. Bayer B. Herndl G. J. Sintes E. (2019). Niche differentiation of aerobic and anaerobic ammonia oxidizers in a high latitude deep oxygen minimum zone. *Front. Microbiol.* 10:2141. 10.3389/fmicb.2019.02141 31572345 PMC6753893

[B96] Mucko M. Bosak S. Casotti R. Balestra C. Ljubešić Z. (2018). Winter picoplankton diversity in an oligotrophic marginal sea. *Mar. Genomics* 42 14–24. 10.1016/j.margen.2018.09.002 30249373

[B97] Müller-Niklas G. Stefan S. Kaltenböck E. Herndl G. J. (1994). Organic content and bacterial metabolism in amorphous aggregations of the northern Adriatic Sea. *Limnol. Oceanogr.* 39 58–68. 10.4319/lo.1994.39.1.0058

[B98] Newton R. J. Griffin L. E. Bowles K. M. Meile C. Gifford S. Givens C. E. (2010). Genome characteristics of a generalist marine bacterial lineage. *ISME J.* 4 784–798. 10.1038/ismej.2009.150 20072162

[B99] Nikaido H. Takatsuka Y. (2009). Mechanisms of RND multidrug efflux pumps. *Biochim. Biophys. Acta* 1794 769–781. 10.1016/j.bbapap.2008.10.004 19026770 PMC2696896

[B100] Nishida I. Murata N. (1996). Chilling sensitivity in plants and cyanobacteria: the crucial contribution of membrane lipids. *Annu. Rev. Plant Biol.* 47 541–568. 10.1146/annurev.arplant.47.1.541 15012300

[B101] Obiol A. Giner C. R. Sánchez P. Duarte C. M. Acinas S. G. Massana R. (2020). A metagenomic assessment of microbial eukaryotic diversity in the global ocean. *Mol. Ecol. Resour.* 20 718–731. 10.1111/1755-0998.13147 32065492

[B102] Oh H.-M. Kwon K. K. Kang I. Kang S. G. Lee J.-H. Kim S.-J. (2010). Complete genome sequence of “*Candidatus* Puniceispirillum marinum” IMCC1322, a representative of the SAR116 clade in the *Alphaproteobacteria*. *J. Bacteriol.* 192 3240–3241. 10.1128/jb.00347-10 20382761 PMC2901696

[B103] Oliveros J. C. (2015). *Venny*. *An Interactive Tool for Comparing Lists with Venn’s Diagrams.* Available at: https://bioinfogp.cnb.csic.es/tools/venny/index.html (accessed September 02, 2020).

[B104] Paliaga P. Felja I. Ušić U. Ivančić I. Najdek M. (2017). Accumulation and persistence of sewage and fish cannery pollution in coastal sediments (northern Adriatic Sea). *J. Soils Sediments* 17 1751–1766. 10.1007/s11368-016-1649-1

[B105] Pimenta A. Racher K. Jamieson L. Blight M. Holland I. (2005). Mutations in HlyD, part of the type 1 translocator for hemolysin secretion, affect the folding of the secreted toxin. *J. Bacteriol.* 187 7471–7480. 10.1128/jb.187.21.7471-7480.2005 16237030 PMC1272971

[B106] Piredda R. Tomasino M. D’erchia A. Manzari C. Pesole G. Montresor M. (2016). Diversity and temporal patterns of planktonic protist assemblages at a Mediterranean Long Term Ecological Research site. *FEMS Microbiol. Ecol.* 93:fiw200. 10.1093/femsec/fiw200 27677681

[B107] Poindexter J. S. (1964). Biological properties and classification of the Caulobacter group. *Bacteriol. Rev.* 28 231–295. 10.1128/mmbr.28.3.231-295.1964 14220656 PMC441226

[B108] Powell S. Forslund K. Szklarczyk D. Trachana K. Roth A. Huerta-Cepas J. (2014). eggNOG v4.0: nested orthology inference across 3686 organisms. *Nucleic Acids Res.* 42 D231–D239. 24297252 10.1093/nar/gkt1253 PMC3964997

[B109] Preston C. M. Durkin C. A. Yamahara K. M. (2019). DNA metabarcoding reveals organisms contributing to particulate matter flux to abyssal depths in the North East Pacific ocean. *Deep Sea Res. II Top. Stud. Oceanogr.* 173:104708 10.1016/j.dsr2.2019.104708

[B110] Rath J. Wu K. Y. Herndl G. J. DeLong E. F. (1998). High phylogenetic diversity in a marine-snow-associated bacterial assemblage. *Aquat. Microb. Ecol.* 14 261–269. 10.3354/ame014261

[B111] Reddy V. S. Shlykov M. A. Castillo R. Sun E. I. Saier M. H. Jr. (2012). The major facilitator superfamily (MFS) revisited. *FEBS J.* 279 2022–2035. 10.1111/j.1742-4658.2012.08588.x 22458847 PMC3425384

[B112] Riley J. Sanders R. Marsay C. Le Moigne F. A. Achterberg E. P. Poulton A. J. (2012). The relative contribution of fast and slow sinking particles to ocean carbon export. *Glob. Biogeochem. Cycles* 26:GB1026.

[B113] RStudio-Team, (2015). *RStudio: Integrated Development for R.* Boston, MA: RStudio.

[B114] Saier M. H. Jr. Beatty J. T. Goffeau A. Harley K. T. Heijne W. Huang S.-C. (1999). The major facilitator superfamily. *J. Mol. Microbiol. Biotechnol.* 1 257–279. 10943556

[B115] Salazar G. Cornejo-Castillo F. M. Borrull E. Díez-Vives C. Lara E. Vaqué D. (2015). Particle-association lifestyle is a phylogenetically conserved trait in bathypelagic prokaryotes. *Mol. Ecol.* 24 5692–5706. 10.1111/mec.13419 26462173

[B116] Shanks A. L. Reeder M. L. (1993). Reducing microzones and sulfide production in marine snow. *Mar. Ecol. Prog. Ser.* 96 43–47. 10.3354/meps096043

[B117] Siddaramappa S. Viswanathan V. Thiyagarajan S. Narjala A. (2018). Genomewide characterisation of the genetic diversity of carotenogenesis in bacteria of the order *Sphingomonadales*. *Microb. Genom.* 4:e000172. 10.1099/mgen.0.000172 PMC5989583 29620507

[B118] Simon H. M. Smith M. W. Herfort L. (2014). Metagenomic insights into particles and their associated microbiota in a coastal margin ecosystem. *Front. Microbiol.* 5:466. 10.3389/fmicb.2014.00466 25250019 PMC4155809

[B119] Simon M. Grossart H.-P. Schweitzer B. Ploug H. (2002). Microbial ecology of organic aggregates in aquatic ecosystems. *Aquat. Microb. Ecol.* 28 175–211. 10.3354/ame028175

[B120] Siokou-Frangou I. Christaki U. Mazzocchi M. G. Montresor M. Ribera, d’Alcalà M. (2010). Plankton in the open Mediterranean Sea: a review. *Biogeosciences* 7 1543–1586. 10.5194/bg-7-1543-2010

[B121] Smith M. W. Zeigler Allen L. Allen A. E. Herfort L. Simon H. M. (2013). Contrasting genomic properties of free-living and particle-attached microbial assemblages within a coastal ecosystem. *Front. Microbiol.* 4:120. 10.3389/fmicb.2013.00120 23750156 PMC3668451

[B122] Sohm J. A. Ahlgren N. A. Thomson Z. J. Williams C. Moffett J. W. Saito M. A. (2016). Co-occurring *Synechococcus* ecotypes occupy four major oceanic regimes defined by temperature, macronutrients and iron. *ISME J.* 10 333–345. 10.1038/ismej.2015.115 26208139 PMC4737926

[B123] Son K. Brumley D. R. Stocker R. (2015). Live from under the lens: exploring microbial motility with dynamic imaging and microfluidics. *Nat. Rev. Microbiol.* 13 761–775. 10.1038/nrmicro3567 26568072

[B124] Soo R. M. Skennerton C. T. Sekiguchi Y. Imelfort M. Paech S. J. Dennis P. G. (2014). An expanded genomic representation of the phylum Cyanobacteria. *Genome Biol. Evol.* 6 1031–1045. 10.1093/gbe/evu073 24709563 PMC4040986

[B125] Spring S. Scheuner C. Göker M. Klenk H.-P. (2015). A taxonomic framework for emerging groups of ecologically important marine gammaproteobacteria based on the reconstruction of evolutionary relationships using genome-scale data. *Front. Microbiol.* 6:281. 10.3389/fmicb.2015.00281 25914684 PMC4391266

[B126] Steiner P. A. Sintes E. Simó R. De Corte D. Pfannkuchen D. M. Ivančić I. (2019). Seasonal dynamics of marine snow-associated and free-living demethylating bacterial communities in the coastal northern Adriatic Sea. *Environ. Microbiol. Rep.* 11 699–707. 10.1111/1758-2229.12783 31286686 PMC6771949

[B127] Stocker R. (2012). Marine microbes see a sea of gradients. *Science* 338 628–633. 10.1126/science.1208929 23118182

[B128] Stuart R. K. Brahamsha B. Busby K. Palenik B. (2013). Genomic island genes in a coastal marine *Synechococcus* strain confer enhanced tolerance to copper and oxidative stress. *ISME J.* 7 1139–1149. 10.1038/ismej.2012.175 23344240 PMC3660668

[B129] Summers M. M. Katz S. Allen E. E. Rouse G. W. (2013). Association of rhizobia with a marine polychaete. *Environ. Microbiol. Rep.* 5 492–498. 10.1111/1758-2229.12043 23864561

[B130] Sun J. Mausz M. A. Chen Y. Giovannoni S. J. (2019). Microbial trimethylamine metabolism in marine environments. *Environ. Microbiol.* 21 513–520. 10.1111/1462-2920.14461 30370577

[B131] Takahashi-Íñiguez T. Aburto-Rodríguez N. Vilchis-González A. L. Flores M. E. (2016). Function, kinetic properties, crystallization, and regulation of microbial malate dehydrogenase. *J. Zhejiang Univ. Sci. B* 17 247–261. 10.1631/jzus.b1500219

[B132] Tinta T. Vojvoda J. Mozetič P. Talaber I. Vodopivec M. Malfatti F. (2015). Bacterial community shift is induced by dynamic environmental parameters in a changing coastal ecosystem (northern Adriatic, northeastern Mediterranean S ea)–a 2-year time-series study. *Environ. Microbiol.* 17 3581–3596. 10.1111/1462-2920.12519 24903068

[B133] Todd J. D. Rogers R. Li Y. G. Wexler M. Bond P. L. Sun L. (2007). Structural and regulatory genes required to make the gas dimethyl sulfide in bacteria. *Science* 315 666–669. 10.1126/science.1135370 17272727

[B134] Trisolino P. di Sarra A. Anello F. Bommarito C. Di Iorio T. Meloni D. (2018). A long-term time series of global and diffuse photosynthetically active radiation in the Mediterranean: interannual variability and cloud effects. *Atmos. Chem. Phys.* 18 7985–8000. 10.5194/acp-18-7985-2018

[B135] Turner J. T. (2002). Zooplankton fecal pellets, marine snow and sinking phytoplankton blooms. *Aquat. Microb. Ecol.* 27 57–102. 10.3354/ame027057

[B136] Turner J. T. (2015). Zooplankton fecal pellets, marine snow, phytodetritus and the ocean’s biological pump. *Prog. Oceanogr.* 130 205–248. 10.1016/j.pocean.2014.08.005

[B137] van der Jagt H. Friese C. Stuut J. B. W. Fischer G. Iversen M. H. (2018). The ballasting effect of Saharan dust deposition on aggregate dynamics and carbon export: aggregation, settling, and scavenging potential of marine snow. *Limnol. Oceanogr.* 63 1386–1394. 10.1002/lno.10779

[B138] Vojvoda J. Lamy D. Sintes E. Garcia J. A. Turk V. Herndl G. J. (2014). Seasonal variation in marine-snow-associated and ambient-water prokaryotic communities in the northern Adriatic Sea. *Aquat. Microb. Ecol.* 73 211–224. 10.3354/ame01718

[B139] Wagner-Döbler I. Biebl H. (2006). Environmental biology of the marine *Roseobacter* lineage. *Annu. Rev. Microbiol.* 60 255–280. 16719716 10.1146/annurev.micro.60.080805.142115

[B140] Ward C. S. Yung C.-M. Davis K. M. Blinebry S. K. Williams T. C. Johnson Z. I. (2017). Annual community patterns are driven by seasonal switching between closely related marine bacteria. *ISME J.* 11 1412–1422. 10.1038/ismej.2017.4 28234350 PMC5437356

[B141] Wiegand S. Jogler M. Boedeker C. Pinto D. Vollmers J. Rivas-Marín E. (2020). Cultivation and functional characterization of 79 planctomycetes uncovers their unique biology. *Nat. Microbiol.* 5 126–140. 10.1038/s41564-019-0588-1 31740763 PMC7286433

[B142] Yilmaz P. Kottmann R. Field D. Knight R. Cole J. R. Amaral-Zettler L. (2011). Minimum information about a marker gene sequence (MIMARKS) and minimum information about any (x) sequence (MIxS) specifications. *Nat. Biotechnol.* 29 415–420. 21552244 10.1038/nbt.1823 PMC3367316

[B143] Yilmaz P. Yarza P. Rapp J. Z. Glöckner F. O. (2016). Expanding the world of marine bacterial and archaeal clades. *Front. Microbiol.* 6:1524. 10.3389/fmicb.2015.01524 26779174 PMC4705458

[B144] Yung C. M. Vereen M. K. Herbert A. Davis K. M. Yang J. Kantorowska A. (2015). Thermally adaptive tradeoffs in closely related marine bacterial strains. *Environ. Microbiol.* 17 2421–2429. 10.1111/1462-2920.12714 25403257

[B145] Yung C.-M. Ward C. S. Davis K. M. Johnson Z. I. Hunt D. E. (2016). Insensitivity of diverse and temporally variable particle-associated microbial communities to bulk seawater environmental parameters. *Appl. Environ. Microbiol.* 82 3431–3437. 10.1128/aem.00395-16 27037125 PMC4959251

[B146] Zaccone R. Caruso G. Calı C. (2002). Heterotrophic bacteria in the northern Adriatic Sea: seasonal changes and ectoenzyme profile. *Mar. Environ. Res.* 54 1–19. 10.1016/s0141-1136(02)00089-2 12148942

[B147] Zhang H. Liu Y. Nie X. Liu L. Hua Q. Zhao G.-P. (2018). The cyanobacterial ornithine–ammonia cycle involves an arginine dihydrolase. *Nat. Chem. Biol.* 14 575–581. 10.1038/s41589-018-0038-z 29632414

