# Supplementary figures and images for: Functional Seasonality of Free-Living and Particle-Associated Prokaryotic Communities in the Coastal Adriatic Sea
Source: Front Microbiol. 2020 Nov 16;11:584222. doi: 10.3389/fmicb.2020.584222 (PMC7701263; doi:10.3389/fmicb.2020.584222)

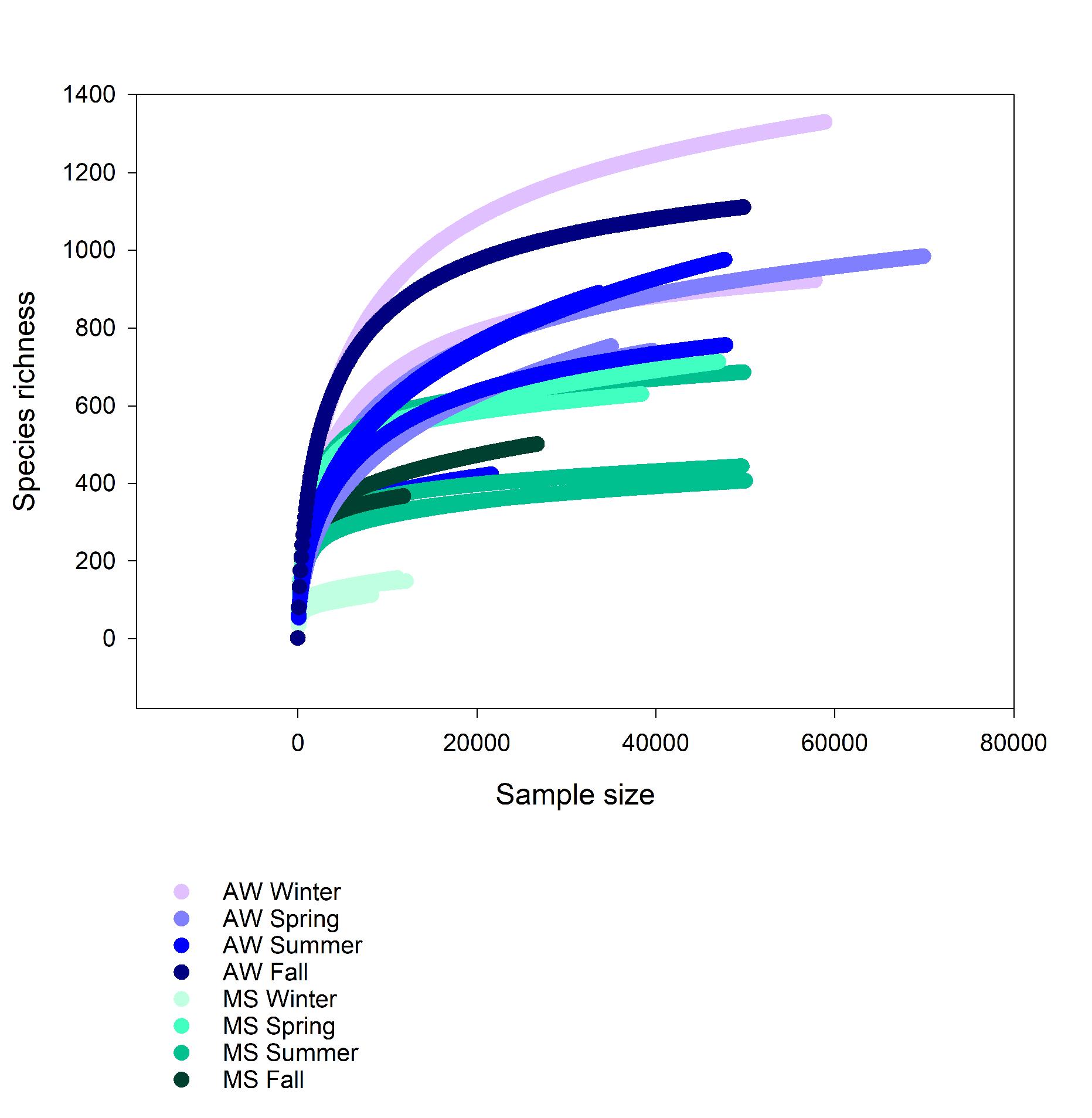

Supplement: Supplementary Figure 1 — Rarefaction curves of amplicon sequences normalized to the sample with smallest number of sequences (08 February 2016 marine snow). [file Image_1.JPEG]

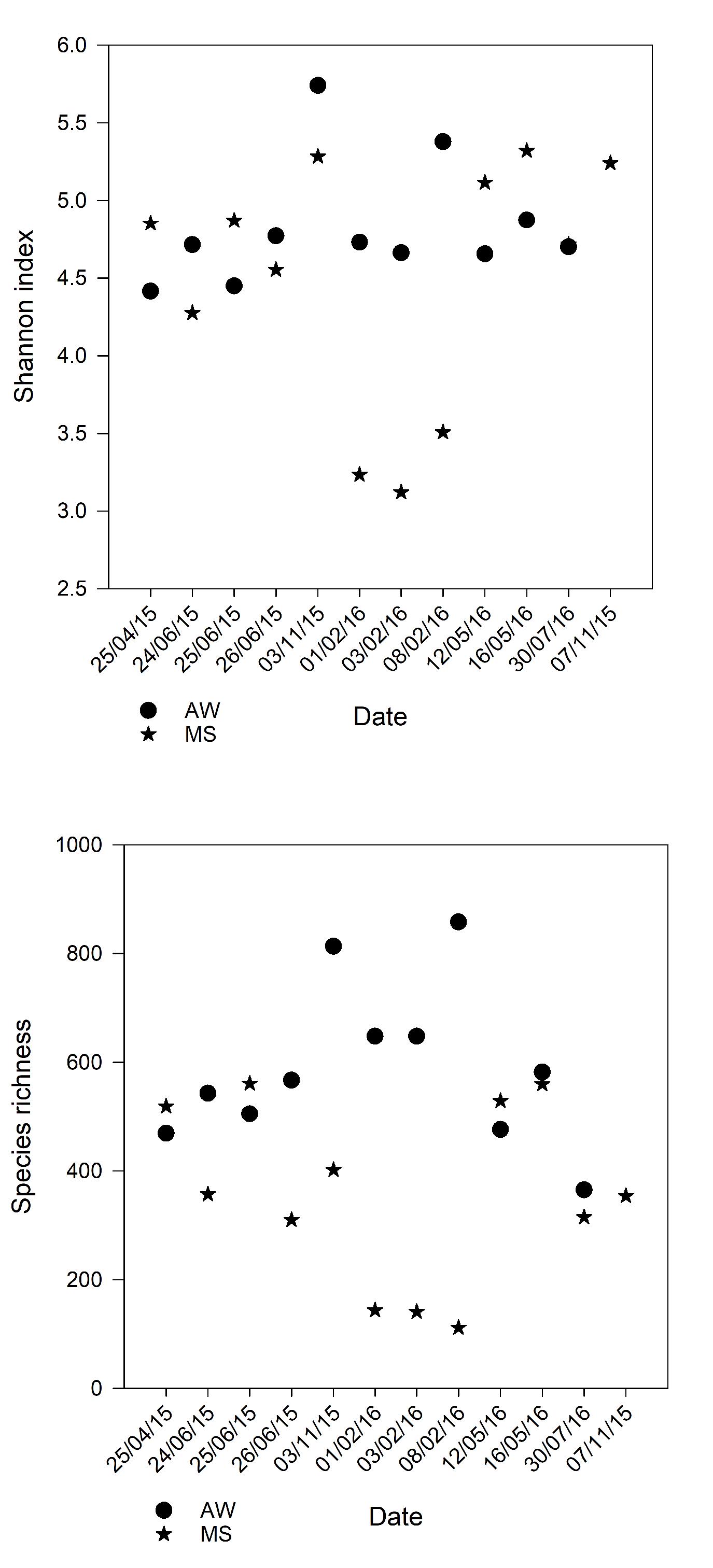

Supplement: Supplementary Figure 2 — Shannon diversity index (A) and species richness (number of taxa; B) of rarified prokaryotic communities in different sampling days based 16S rRNA amplicon sequence variant (ASV) analysis. Symbols indicate ambient water (AW) and marine snow (MS) habitats. [file Image_2.JPEG]

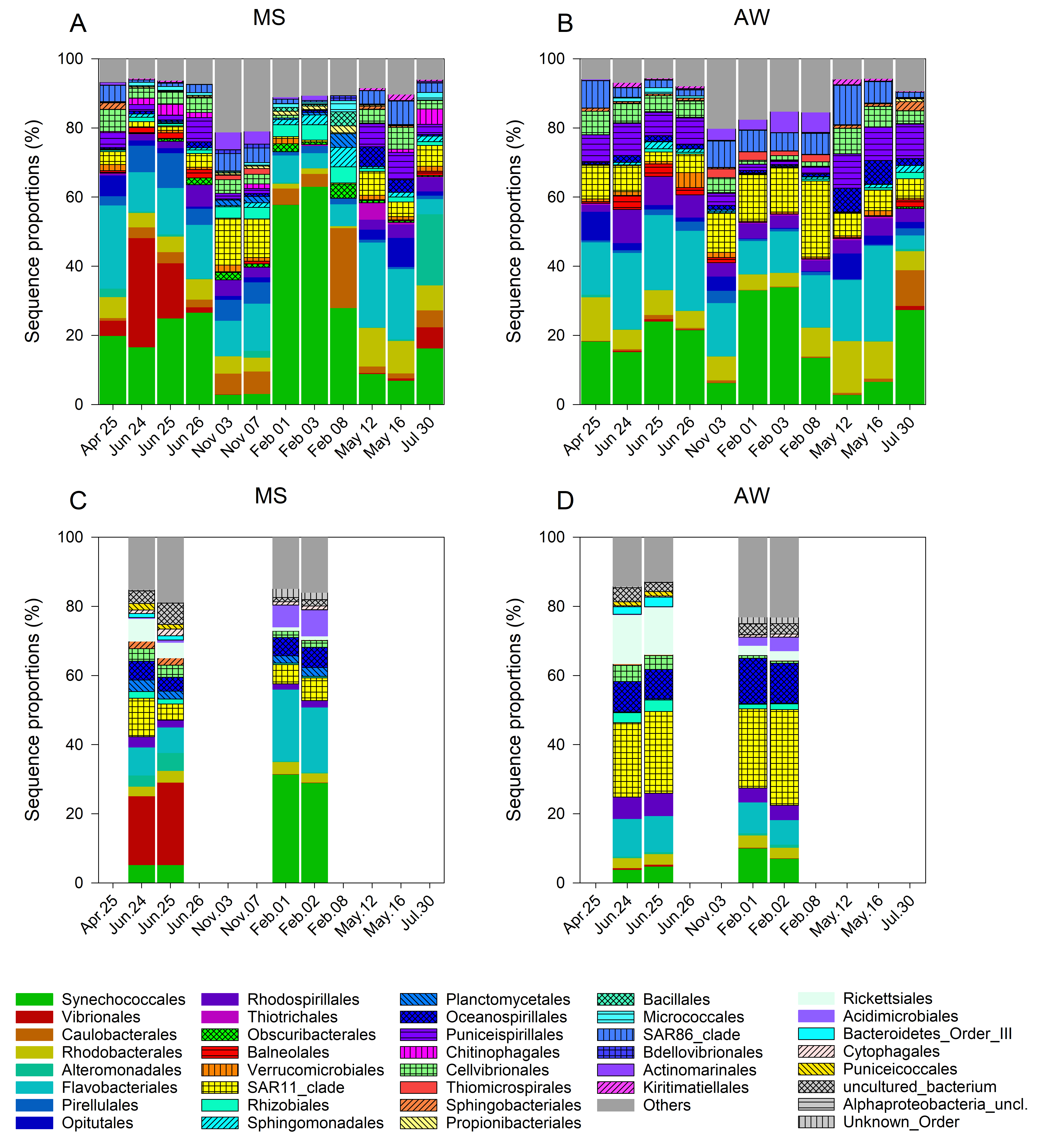

Supplement: Supplementary Figure 3 — Contribution of the 30 most abundant (A) MS-associated (MS) and (B) ambient water (AW) prokaryotic orders to the communities based on variance-stabilized 16S rRNA amplicon sequences and the 20 most abundant (C) MS-associated (MS) and (D) AW prokaryotic orders based on variance stabilized 16S rRNA sequences extracted from the metagenomes. Less abundant prokaryotic orders are grouped under “Others.” Samples from April to November were taken in 2015 and samples from February to July were taken in 2016. [file Image_3.JPEG]

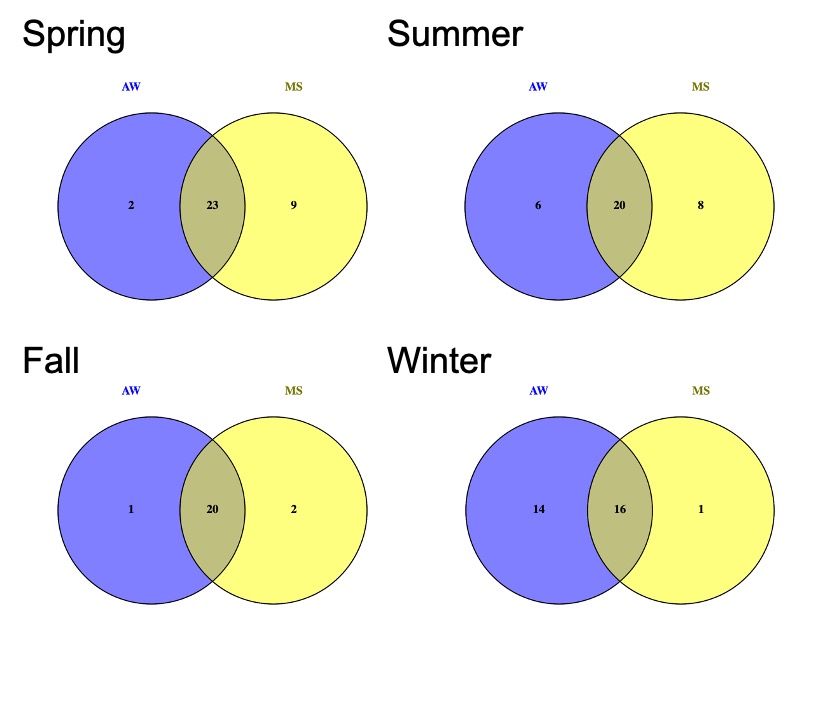

Supplement: Supplementary Figure 4 — Venn diagrams showing the number of prokaryotic classes occurring in the ambient water (AW; blue), the marine snow (MS; yellow) and in both (overlapping circles) each season. [file Image_4.JPEG]

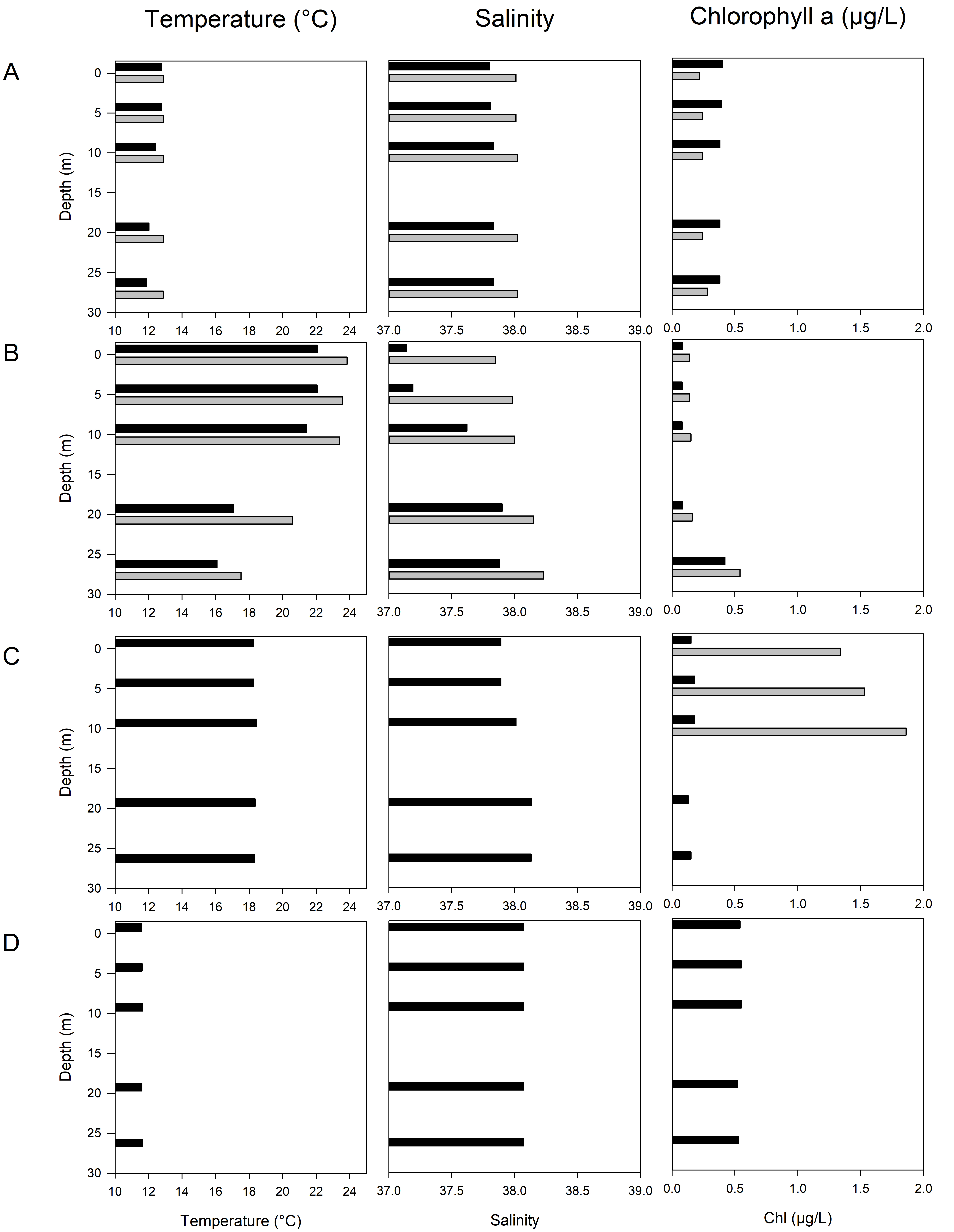

Supplement: Supplementary Figure 5 — Temperature, salinity and chlorophyll a profiles at station RV001 in (A) spring (14/04/15 black bar and 06/04/16 grey bar), (B) summer (26/06/15 black bar and 19/07/16 gray bar), (C) fall (26/10/15 black bar and 09/11/15 gray bar) and (D) winter (20/01/16). [file Image_5.JPEG]

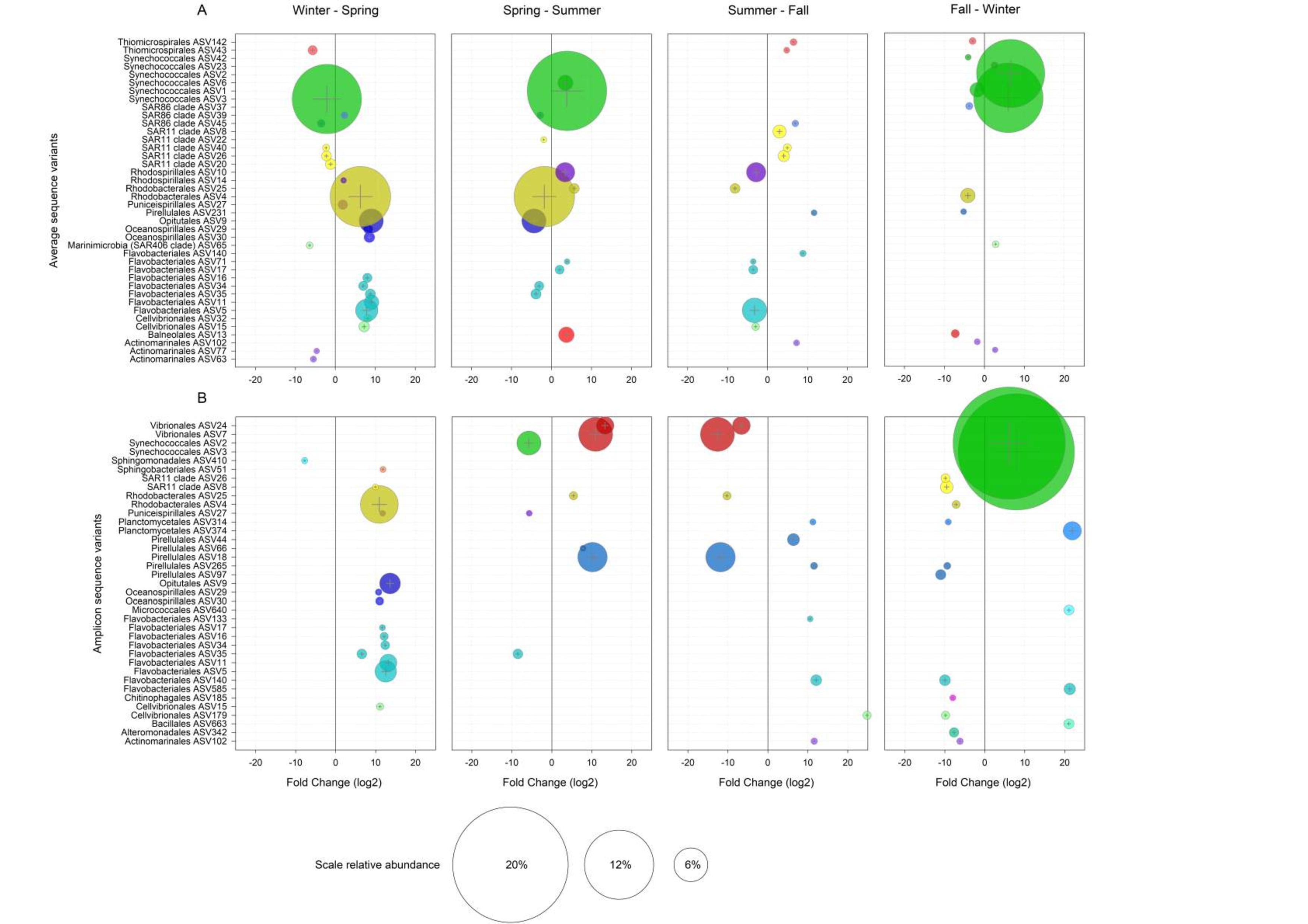

Supplement: Supplementary Figure 6 — Amplicon sequence variants (ASVs) with a relative abundance > 1% and a log2 fold change > 1 significantly (p < 0.05) enriched in one of two consecutive seasons in (A) the ambient water and (B) in marine snow (MS). Bubble size represents the relative abundance and colors indicate the assigned taxonomy at the order level. [file Image_6.JPEG]
